# Divergent uranium- *versus* phosphorus-based reduction of Me_3_SiN_3_ with steric modification of phosphido ligands†

**DOI:** 10.1039/d0sc02261f

**Published:** 2020-05-27

**Authors:** Robert J. Ward, Pokpong Rungthanaphatsophon, Iker del Rosal, Steven P. Kelley, Laurent Maron, Justin R. Walensky

**Affiliations:** Department of Chemistry, University of Missouri Columbia MO 65211 USA walenskyj@missouri.edu; Universite de Toulouse, CNRS, INSA, UPS, UMR, UMR 5215 LPCNO 135 Avenue de Ranguiel 31077 Toulouse France

## Abstract

We describe an example of a two-electron metal- and ligand-based reduction of Me_3_SiN_3_ using uranium(iv) complexes with varying steric properties. Reaction of (C_5_Me_5_)_2_U(CH_3_)[P(SiMe_3_)(Ph)] with Me_3_SiN_3_ produces the imidophosphorane complex, (C_5_Me_5_)_2_U(CH_3_)[N=P(SiMe_3_)_2_(Ph)] through oxidation of phosphorus. However, a similar reaction with a more sterically encumbering phosphido ligand, (C_5_Me_5_)_2_U(CH_3_)[P(SiMe_3_)(Mes)] forms the U(iv) complex, (C_5_Me_5_)_2_U[*κ*^2^-(*N*,*N*)–N(SiMe_3_)P(Mes)N(SiMe_3_)]. In probing the mechanism of this reaction, a U(vi) bis(imido) complex, (C_5_Me_5_)_2_U(=NSiMe_3_){=N[P(SiMe_3_)(Mes)]} was isolated. DFT calculations show an intramolecular reductive cycloaddition reaction leads to the formation of the U(iv) bis(amido)phosphane from the U(vi) bis(imido) complex. This is a rare example of the isolation of a reaction intermediate in *f* element chemistry.

## Abstract 2

We describe an example of a two-electron metal- and ligand-based reduction of Me_3_SiN_3_ using uranium(iv) complexes with varying steric properties. With uranium-based reduction, a U(vi) intermediate is isolated.

## Graphical abstract

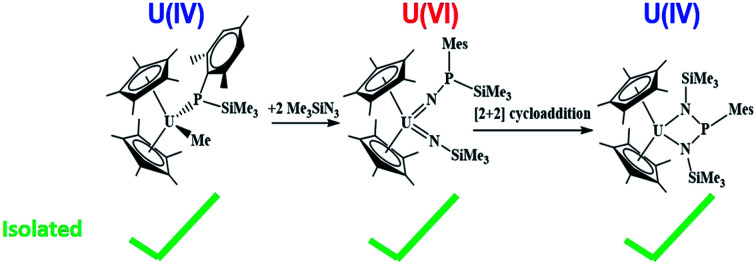

## Introduction

Many important reactions involve metal-based catalysis. Suzuki coupling,^1^ the Heck reaction,^2^ Wilkinson's catalyst,^3^ and many other catalytic cycles require oxidation and reduction reactions to work in tandem. These are all transition metal-based catalysts since two-electron redox couples are readily available. Within the 5*f* block,^4^ uranium is one of the only metals for which a two-electron redox couple is facile, and while oxidation is relatively easy to achieve, reduction is rarely observed without the use of an external reducing agent.^5^

**Scheme 1:**
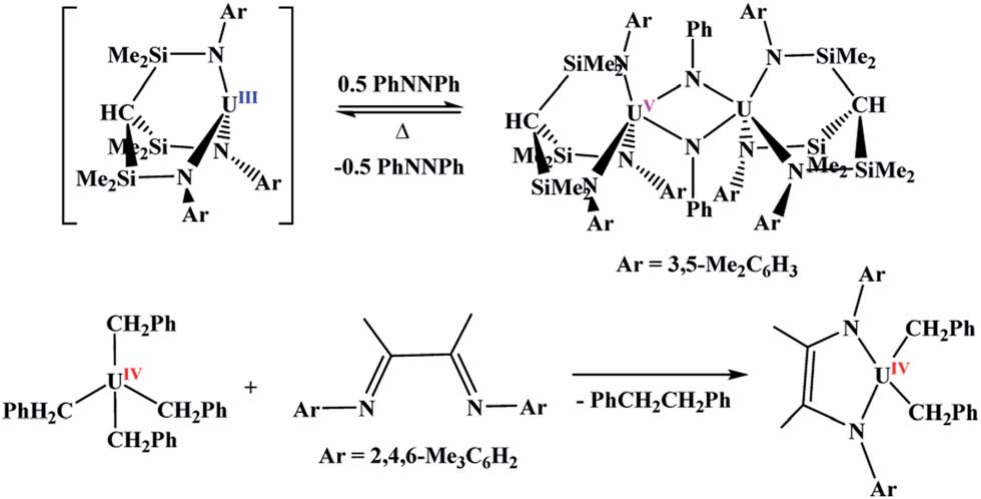
Examples of two-electron oxidative and reductive reactions in uranium chemistry.

With uranium, two-electron metal-based oxidation is achieved most readily with U(iii) either through using two equivalents of the U(iii) starting material to form two U(iv) species, or direct oxidation to U(v). However, in all examples of oxidative chemistry with the actinides, a subsequent reductive step is rarely observed. Recently, the Liddle group reported the oxidation of U(iii) to U(v) using azobenzene,^6^Scheme 1. Under reduced pressure and gentle heating, Liddle's U(v) dimer undergoes reduction to the U(iii) starting material. This is the only example in which the oxidative and reductive steps have been isolated in which the oxidation state of the metal changes. The other reductive chemistry seen with the actinides is with redox-active ligands,^7,8^Scheme 1,^9,10^ with no observed change in the oxidation state of the metal. Since two-electron metal-based reactions are important in catalysis^11,12^ and small molecule activation,^13,14^ it is of interest to have a greater understanding of both the oxidative and reductive processes.

One of the most investigated substrates for interrogating U(iii) reaction chemistry has been organic azides.^15^ This two-electron reduction typically is done by the metal centre to form a U(v) imido. There are limited examples of U(iv) oxidation to U(vi),^16–23^ all of them involving oxo- or imido-delivering agents. Our group has taken the approach of examining the reactivity of An(iv), An = Th, U, complexes with soft donor ligands, such as phosphorus, which also has a rich chemistry with organic azides.^24^ These complexes impart a mismatch between the hard, electropositive Lewis acidic actinide centre, and the soft Lewis basic nature of phosphorus, and have been shown to afford unpredictable and unusual chemistry.^25–34^ We recently showed that the U(iii) complex, (C_5_Me_5_)_2_U(THF)[P(SiMe_3_)(Mes)] reacts with Me_3_SiN_3_ to form the U(vi) bis(imido) complex, (C_5_Me_5_)_2_U(=NSiMe_3_)_2_.^35^ In order to prevent the formation of the bis(imido) complex, here, we used the mixed phosphido–methyl complexes (C_5_Me_5_)_2_U(CH_3_)[P(SiMe_3_)(R)], R = C_6_H_5_ (Ph), **1**; 2,4,6-Me_3_C_6_H_2_ (Mes), **2**. The mesityl complex was recently shown to react unusually with ^*t*^BuNC through a series of cascade reactions to form an α-diimine,^36^ however, isocyanides do not undergo redox chemistry akin to organic azides such as Me_3_SiN_3_. Complexes **1** and **2** differ only in the steric properties of the *R* group associated with the phosphido ligand. Herein, we demonstrate that the steric properties of the aryl of the phosphido ligand play an integral role in product formation, including the isolation of a U(vi) intermediate, which subsequently reduces to U(iv) to form the final product. To our knowledge, there are no examples in *f* element chemistry in which an intermediate has been isolated and characterized.

## Results and discussion

The phosphido–methyl complexes, (C_5_Me_5_)_2_U(CH_3_)[P(SiMe_3_)(Ph)], **1**, and (C_5_Me_5_)_2_U(CH_3_)[P(SiMe_3_)(Mes)], **2**,^36^ were prepared in high yield from the reaction of (C_5_Me_5_)_2_U(CH_3_)(i) with K[P(SiMe_3_)(R)], R = Ph or Mes, respectively, eqn (1). Both these complexes are brown-black in colour as compared to their dark red starting materials. No ^31^P NMR resonances were found from −5000 to +5000 ppm. While spectroscopic and analytical characterization of **1** was done, despite numerous attempts, the solid-state structure could not be obtained. However, due to the similarity in the ^1^H NMR spectra of **1** and **2**, in addition to the reaction chemistry reported herein, we surmise that these two are structurally comparable.1
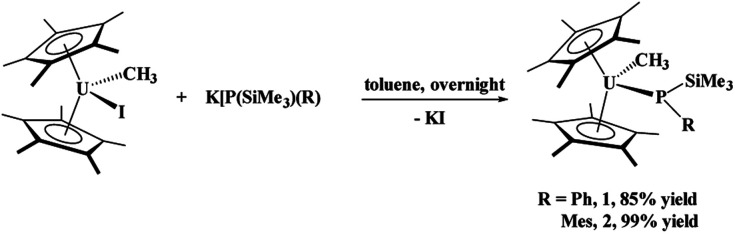


To probe the reactivity of these complexes, an organic azide was used since nitrogen is a hard Lewis base, compared to phosphorus, and organic azides have been shown to have both insertion^37–40^ and reductive reactivity.^38,41^ We specifically used Me_3_SiN_3_ since it does not typically insert into actinide–carbon bonds,^37^ and thus the reactivity should occur only at the uranium–phosphido bond. Reaction of (C_5_Me_5_)_2_U(CH_3_)[P(SiMe_3_)(Ph)], **1**, with two equivalents of Me_3_SiN_3_, eqn (2), does not produce a colour change as the solution remains black, but effervescing was observed instantaneously. The low yield (18%) reported is based on the crystalline product, but the crude NMR spectrum shows the formation of one product. The low yield is attributed to high solubility of the complex in hydrocarbon solvents. Even with excess amount of Me_3_SiN_3_, the same product is obtained. The ^1^H NMR spectrum revealed a single product with a resonance at −194.4 ppm, indicative of a methyl group still coordinated to the paramagnetic uranium center.^42–44^ This resonance shifts slightly compared to the starting material at −190.2 ppm. In addition, the ^1^H NMR spectrum showed the (C_5_Me_5_)^1−^ resonance at −1.35 ppm, but another resonance integrating to 18 protons was detected at 10.3 ppm. A signal in the ^31^P NMR spectrum at 518.0 ppm was located.2
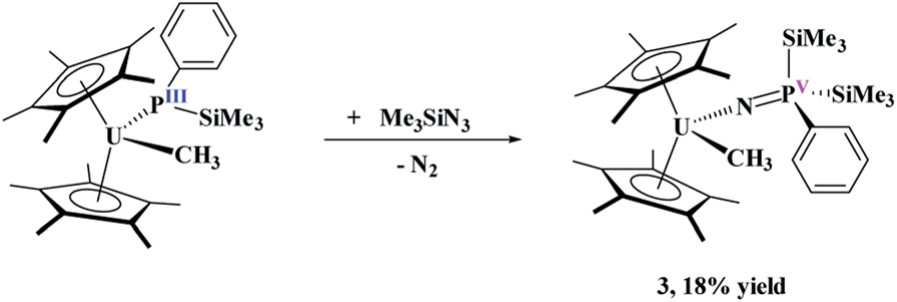


**Fig. 1:**
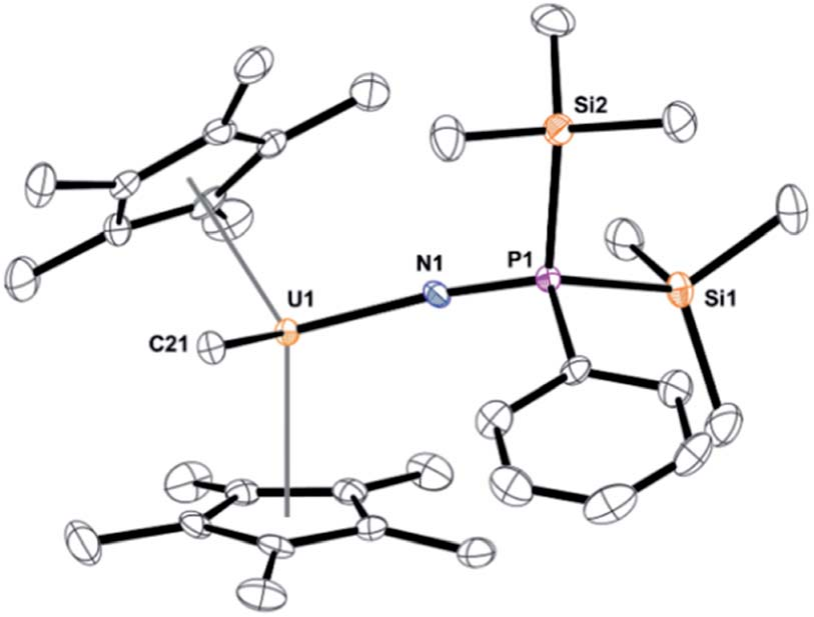
Thermal ellipsoid plot of **3** shown at the 50% probability level. The hydrogen atoms have been omitted for clarity.

Dark yellow crystals, suitable for X-ray crystallography analysis, were grown from a saturated toluene solution at −25 °C. The solid-state structure, Fig. 1, identified the product as (C_5_Me_5_)_2_U(CH_3_)[N=P(SiMe_3_)_2_(Ph)], **3**. Complex **3** fits the NMR spectroscopy data with two trimethylsilyl groups coordinated to phosphorus and a methyl still bound to uranium. Complex **3** has a pseudo-tetrahedral arrangement with two (C_5_Me_5_)^1−^ ligands as well as the methyl and the newly formed imidophosphorane ligand. The U(iv)-nitrogen bond distance of 2.098(3) Å is similar to uranium ketimide, (N=CR_2_)^1−^, complexes. For example, (C_5_H_5_)_3_U[N=C(Me)CHPMePh_2_]^45^ and (C_5_Me_5_)_2_U[N=C(Ph)CH_2_Ph]_2_ (ref. 46) have U–N bond distances of 2.06(1) Å and 2.184(3) Å, respectively. A bond distance of 2.07(2) Å was observed in Gilje's imidophosphorane complex, (C_5_H_5_)_3_U(N=PPh_3_).^47^ The U–N–P bond angle of 171.6(2)° in 3 is identical to the 172(1)° in Gilje's compound. Additionally, the U(iv) tetrakis(imidophosphorane) complex, U[N=P(pip)_3_]_4_, pip = piperidinyl, was recently reported with average U–N bond lengths of 2.19(5) Å.^18^ A thorium complex, (1,2,4-^*t*^Bu_3_C_5_H_2_)_2_Th(N_3_)[N=P(2,4,6-^*t*^Bu_3_C_6_H_2_)], with a similar Th–N (iminophosphino) bond length of 2.273(9) Å, has also been recently reported.^25^

Formation of **3** involves the insertion of an imido unit into the uranium–phosphorus bond, with a silyl migration to phosphorus. Overall, this is a two-electron reduction of the azide to an imido moiety with simultaneous oxidation of the phosphorus from +3 to +5. We could find only one example of similar reactivity in the literature using a titanium phosphine complex with dinitrogen.^48^ In that case, the reduced dinitrogen oxidizes the phosphorus but without ligand addition to phosphorus. In fact, silylated iminophosphanes, (R_3_SiNPR_3_), are common starting materials to form transition metal complexes,^49–52^ however, in no case has the silyl been observed to migrate to phosphorus. We note that recently a mesityl ligand has been observed to migrate from nitrogen to phosphorus.^53^ To our knowledge, no examples of this imidophosphorane, with two trimethylsilyl groups and one phenyl, are known as mixed-substituted ligands are rare.

When the steric properties of the *R* group were increased from phenyl to mesityl, we expected the product to be the same, as was seen with the reactivity with ^*t*^BuNC.^15^ Reaction of **2** with Me_3_SiN_3_ in 1,2-dimethoxyethane (DME) also had no colour change but effervescing was observed, eqn (3). The ^1^H NMR spectrum revealed two (C_5_Me_5_)^1−^ resonances at 10.7 and 5.01 ppm and one SiMe_3_ resonance at −21.2 ppm. However, the resonance for a methyl group coordinated to uranium(iv) was not observed. A resonance in the ^31^P NMR spectrum was found at −180.8 ppm, shifted considerably from that observed in **3**. Dark red crystals in 66% yield were grown from a saturated toluene solution at −25 °C, and the structure was unambiguously identified as (C_5_Me_5_)_2_U{*κ*^2^-(*N*,*N*ʹ)–[N(SiMe_3_)]_2_P(Mes)}, **4**, Fig. 2.3
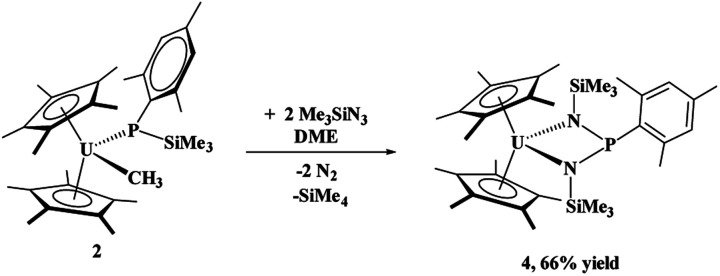


**Fig. 2:**
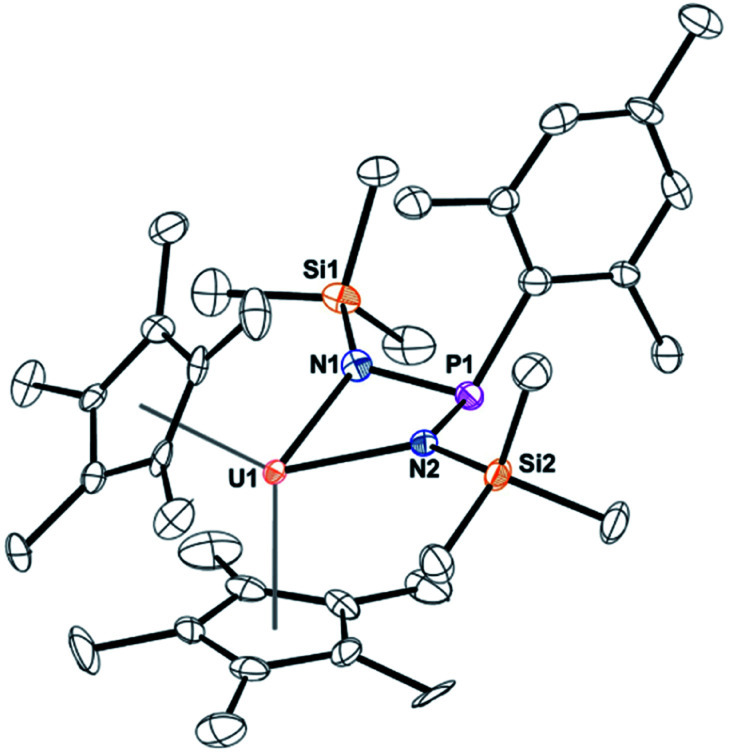
Thermal ellipsoid plot of **4** shown at the 50% probability level. The hydrogen atoms have been omitted for clarity.

The structure of **4** is a uranium(iv) centre with the metallocene ligand framework and a bis(amido)phosphane ligand in a pseudo-tetrahedral environment.^54–56^ The uranium–nitrogen bond distances of 2.277(4) Å compare well with the U–N distances in U(iv) α-diimine complexes, (^Mes^DAB^Me^)_2_U(THF) of 2.251(4)–2.255(4) Å,^57^ or 2.273(2) and 2.3331(2) Å in (C_5_Me_5_)_2_U[*κ*^2^-(*N*,*N*)–N(^*t*^Bu)C=CN(^*t*^Bu)C=N(^*t*^Bu)CH_2_].^36^ The P–N bond distances are 1.734(4) and 1.741(4) Å, much longer than the 1.600(3) Å found in **3**, indicative of a P–N single bond.

The difference in reactivity of **1** and **2** with Me_3_SiN_3_ was examined using DFT calculations (see ESI†). Energetically, the formation of the mesityl analogue of **3**, (C_5_Me_5_)_2_U(CH_3_)[N=P(SiMe_3_)_2_(Mes)], **3Mes**, is similar to the formation of **3**, with the exception of the silyl transfer which is endothermic for **3Mes**. Hence, this indicates the two reaction mechanisms are different from the initial step and do not share similarity. Thus, we then attempted to investigate the formation of **4** more closely. The reaction was conducted at −45 °C for 10 minutes in DME. To our surprise, a diamagnetic ^1^H NMR spectrum was obtained with one (C_5_Me_5_)^1−^ resonance located at 4.67 ppm, and two SiMe_3_ groups at 1.03 and 1.17 ppm. The ^31^P NMR resonance was located at 157 ppm. Upon crystallization from a saturated diethyl ether solution at −45 °C, (C_5_Me_5_)_2_U{=N[P(SiMe_3_)(Mes)]}[=N(SiMe_3_)], **5**, eqn (4), was identified as the product, but in very low yields (<10%). We have found that short reaction times are optimal for the isolation of **5**, otherwise the product progresses to **4**. Despite the low yield, crystalline material can be obtained from the reaction mixture in a reproducible manner. The byproduct, SiMe_4_, was observed in the crude NMR spectra for both **4** and **5**. The formation of **5** using the U^VI/IV^ redox couple is rare^16,18–21^ and nearly all examples involve forming bis(imido)^17,22^ complexes or uranyl functionalization^23,58^ as the U^V/III^ redox is far more common,^59^ especially with azide reduction. Complex **5** is unusual as nearly all bis(imido) actinide complexes have a nitrogen–carbon, silicon, or hydrogen linkage.^15^ The ^1^H NMR spectrum of **5** shows temperature independent paramagnetism, a common feature of U(vi) bis(imido) complexes.^60,61^ When **5** is further stirred at room temperature, the conversion of **4** is observed, eqn (5). While there is precedent for oxidative chemistry with U(iv) complexes to form U(vi) bis(imido) complexes, those complexes do not reduce to U(iv) without addition of H_2_ (ref. 62) or an external reductant.^63^4
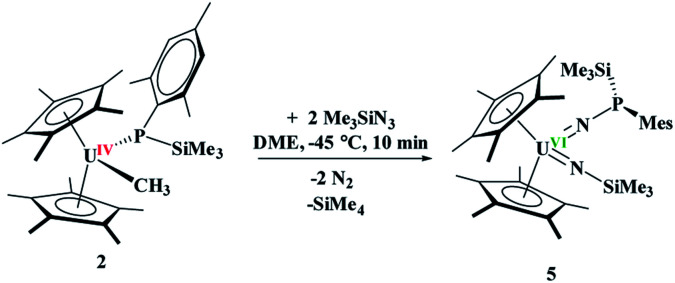
5
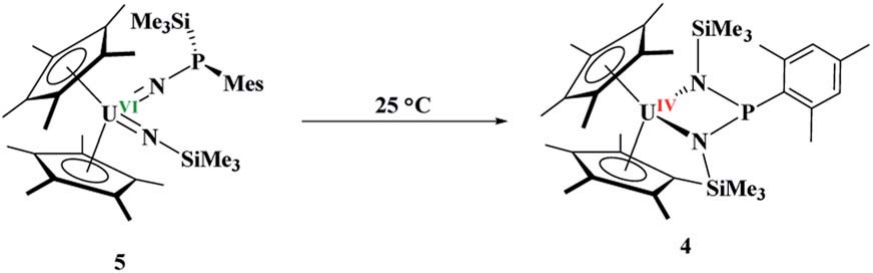


**Fig. 3:**
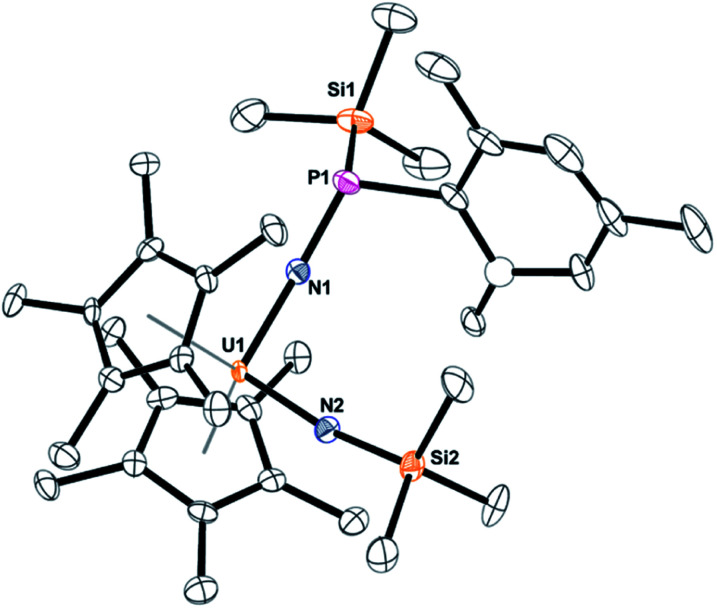
Thermal ellipsoid plot of **5** shown at the 50% probability level. The hydrogen atoms have been omitted for clarity. One carbon atom is shown anisotropically (see ESI†).

The solid-state structure of **5** was determined by X-ray crystallography, Fig. 3. Complex **5** is the first bis(imido) complex with a phosphorus coordinated to nitrogen, but reminiscent of the bis(imido) complex, (C_5_Me_5_)_2_U[=NN=CPh_2_][=N(2,4,6-^*t*^Bu_3_C_6_H_2_)].^17^ The U–N bond distances of 2.00(1) and 1.952(9) Å, compare well to those in (C_5_Me_5_)_2_U[=NN=CPh_2_][=N(SiMe_3_)] of 2.031(6) and 1.987(5) Å. In both complexes, the longer U–N bond distance is the one associated with the phosphorus or nitrogen, respectively. The P(iii)–N bond distance in **5** is 1.67(1) Å is identical to those observed in phosphanamides.^64^

**Fig. 4:**
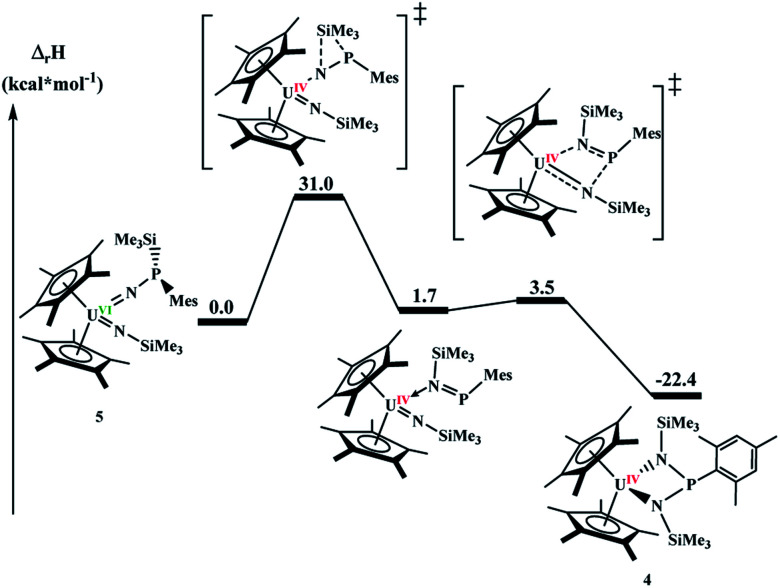
Computed enthalpy profile for the transformation of complex **5** into complex **4**.

Since **4** and **5** are structural isomers, the rearrangement must be intramolecular or solvent-assisted. Density functional theory calculations were performed to provide further insight into this transformation. A plausible reaction mechanism was obtained at the DFT level of theory (B3PW91, Fig. 4).

The reaction begins by the intramolecular transfer (1,2 shift) of the SiMe_3_ moiety on complex **5**. The associated barrier is 31.0 kcal mol^−1^ meaning that the reaction is moderately fast, but high enough that **5** was able to be isolated. Interestingly, the TS is found on the triplet spin potential energy surface (PES), indicating that the reduction of the uranium centre already occurs during the silyl migration (the reaction on the singlet PES. The singlet PES for U(vi) was also computed and found to occur at a similar energy, 29.6 kcal mol^−1^ but yields a less stable intermediate, as shown in ESI†). Following the intrinsic reaction coordinates, the system evolves to the formation of a U(iv) imido-phospho-imino complex, that is only 1.7 kcal mol^−1^ less stable than complex **5**. The latter complex readily undergoes a [2+2] cycloaddition (activation barrier of 1.8 kcal mol^−1^), yielding the final complex **4**. Its formation is exothermic by 22.4 kcal mol^−1^.

With only a small change in the steric properties of phosphido ligands, Me_3_SiN_3_ was reduced by either P(iii) or U(iv). The reasoning behind why one is favoured over another is not completely understood at present, but the calculations suggest that the imidophosphorane is not favourable with the mesityl group, hence an alternate, lower energy pathway, *i.e.* uranium oxidation, is performed instead of phosphorus oxidation. Until now, no examples of uranium oxidation and subsequent reduction were observed in the same reaction, and thus the conversion of **2** to **4***via***5** affords a snapshot of these processes working in concert. Finally, few cycloaddition reactions are known in *f* element chemistry,^36,65–71^ and none of them involve metal-based reduction.

## Conclusions

In summary, we have examined the reactivity of Me_3_SiN_3_ with U(iv) metallocene complexes bearing mixed phosphido–methyl ligands. With a simple change of the aryl group on the phosphido ligand from phenyl to mesityl, the reactivity changed dramatically. With a smaller phenyl group, phosphorus oxidation is observed, and a silyl migration occurs from nitrogen to phosphorus. When the larger mesityl is present, a second azide is reduced. This was shown to proceed through a U(vi) bis(imido) complex followed by rearrangement through silyl transfer *via* a reductive intramolecular [2+2] cycloaddition. While the two-electron reduction of azides is well-known with phosphorus and uranium, we have demonstrated that either can reduce Me_3_SiN_3_ based on the steric properties of the phosphido ligand.

## Conflicts of interest

There are no conflicts to declare.

## Supplementary Material

SC-011-D0SC02261F-s001

SC-011-D0SC02261F-s002
