# Supplementary material for: Divergent uranium- versus phosphorus-based reduction of Me3SiN3 with steric modification of phosphido ligands
Source: Chem Sci. 2020 May 27;11(22):5830–5. doi: 10.1039/d0sc02261f (PMC8159289; doi:10.1039/d0sc02261f)
Supplement: SC-011-D0SC02261F-s001 [file SC-011-D0SC02261F-s001.pdf]

Electronic Supplementary Material (ESI) for Chemical Science.
This journal is © The Royal Society of Chemistry 2020

Supporting Information for

Divergent Uranium- versus Phosphorus-Based Reduction of Me_3SiN_3 with Steric Modification of Phosphido Ligands

Table of Contents

Experimental S2

Table S1. Crystallographic parameters for complexes **3** and **4**. S8

Figure S1. ^{1}H NMR spectrum of **1** in C_6D_6. .... S10

Figure S2. ^{1}H NMR spectrum of **2** in C_6D_6. .... S10

Figure S3. ^{1}H NMR spectrum of **3** in C_6D_6. .... S11

Figure S4. Close-up of the ^{1}H NMR spectrum of **3** in C_6D_6. .... S12

Figure S5. ^{31}P{^{1}H} NMR spectrum of **3** in C_6D_6. .... S12

Figure S6. ^{1}H NMR spectrum of **4** in C_6D_6. .... S13

Figure S7. Close-up of the ^{1}H NMR spectrum of **4** in C_6D_6. .... S14

Figure S8. ^{31}P{^{1}H} NMR spectrum of **4** in C_6D_6. .... S14

Figure S9. ^{1}H NMR spectrum of **5** in C_6D_6. .... S15

Figure S10. ^{13}C NMR spectrum of **5** in C_6D_6.. S15

Figure S11. ^{31}P NMR spectrum of **5** in C_6D_6.. S16

Figure S12. NMR spectra of the conversion of **5** (red) to **4** (blue)………………………….... S16

Computational Details…………………………………………………………………… S17

Figure S13. Computed enthalpy profile for the conversion of **1** into **3** and of **2** to **3**$^{\mathbf{Mes}}$………S18

Figure S14. Computed enthalpy profile for the conversion of **5** into **4** on the singlet spin state potential energy surface……………………………………………………………………...S19

Coordinates for calculated structures………………………………………………………...S20

References S71

Experimental

General consideration. All syntheses were carried out under inert atmosphere of nitrogen using standard Schlenk and glovebox techniques. All glassware used (including NMR tubes) were silylated with Me_3SiCl, at reduced pressure, inside of glass vacuum desiccator. 1,2-dimethoxyethane (Chem Impex) was distilled from a benzophenone-potassium still, stored over activated 4 Å sieves within the inert atmosphere box, and used within 10 days. Azidotrimethylsilane and $KN(SiMe_3)_2$ (Aldrich) were used as received from the supplier. $(C_5Me_5)_2U(CH_3)(Cl)$,[1] $(C_5Me_5)_2U(CH_3)(I)$,[2] and K[P(Mes)($SiMe_3$)][3] were synthesized according to literature procedure. K[P(C_6H_5)($SiMe_3$)] was prepared from HP(C_6H_5)($SiMe_3$)[4] and $KN(SiMe_3)_2$ in toluene. Benzene-d_6 was dried over molecular sieves and degasses with three cycles of freeze-pump-thaw. All ^{1}H and ^{13}C NMR spectrum were collected either on Bruker Avance III 500 or 600 MHZ spectrometer. ^{31}P NMR spectrum were collected on Bruker AVII+ 300MHz spectrometer. ^{1}H and ^{13}C NMR chemical shifts were reported referenced internally to the residue solvent peak at 7.16 ppm (C_6D_5H) and 128.06 ($^{13}C_6D_6$). ^{31}P NMR chemical shifts were reported referenced externally to H_3PO_4 at 0 ppm. All heteronuclear NMR spectra were recorded proton-decoupled. Infrared spectra were recorded as a KBr pellets on Perkin-Elmer Spectrum One FT-IR spectrometer. Elemental analyses were performed by Microanalysis Facility, College of Chemistry, University of California, Berkeley.

Synthesis of $(C_5Me_5)_2U(CH_3)[P(C_6H_5)(SiMe_3)]$, 1. To a scintillation vial charged with $K[P(C_6H_5)(SiMe_3)]$ (40 mg, 0.18 mmol) and toluene (5 mL), $(C_5Me_5)_2U(CH_3)(Cl)$ (100 mg, 0.18 mmol) in toluene (5 mL) was added. The solution was stirred at room temperature overnight, after which, it was filtered through Celite and the solvent was removed *in vacuo* to yield a dark brown solid (107 mg, 85%). ^{1}H NMR (C_6D_6, 600 MHz, 298 K): 11.4 (s, 30H, $C_5(CH_3)_5$, $v_{1/2}$ = 11 Hz), -4.21 (s, 1H, *p*-Ph, $v_{1/2}$ = 14 Hz), -5.35 (s, 2H, *m*-Ph, $v_{1/2}$ = 14 Hz), -14.6 (s, 9H, Si*Me*$_3$, $v_{1/2}$ = 8 Hz), -35.6 (s, 2H, *p*-Ph, $v_{1/2}$ = 38 Hz), -190.2 (s, 3H, CH_3, $v_{1/2}$ = 144 Hz). IR (KBr, cm^{-1}): 2903 (vs), 2858 (s), 2725 (w), 1578 (m), 1473 (m), 1432 (br-m), 1378 (m), 1259 (w), 1242 (s), 1102 (s), 1081 (s), 1022 (s), 889 (w), 837 (vs), 747 (w), 726 (w), 693 (w), 629 (w). Anal. Calcd for $C_{30}H_{47}PSiU$: C, 51.13; H, 6.72. Found: C, 50.67; H, 6.26.

Synthesis of $(C_5Me_5)_2U(CH_3)[P(Mes)(SiMe_3)]$, 2. Complex **2** was prepared in a manner similar to **1** using $(C_5Me_5)_2U(CH_3)I$ (252 mg, 0.39 mmol) in toluene (3 mL) and KP[(Mes)($SiMe_3$)] (102 mg, 0.39 mmol) in toluene (5 mL). Analytically pure sample of **2** was obtained after filtration through Celite and solvent removal (285 mg, 99%). ^{1}H NMR (C_6D_6, 500 MHz, 298 K): δ 9.45 (s, 30H, $C_5(CH_3)_5$, $v_{1/2}$ = 7 Hz), 2.46 (s, 2H, *m*-Mes, , $v_{1/2}$ = 2 Hz), -0.20 (s, 3H, CH_3-*p*-Mes, $v_{1/2}$ = 3 Hz), -6.78 (s, 6H, CH_3-*o*-Mes, , $v_{1/2}$ = 10 Hz), -14.3 (s, 9H, $Si(CH_3)_3$, $v_{1/2}$ = 8 Hz), -125.0 (s, 3H, U-CH_3, $v_{1/2}$ = 50 Hz). IR (KBr, cm^{-1}): 2949 (m), 2908 (s), 2867 (m), 1438 (m), 1376 (m), 1237 (m), 1102 (w), 1044 (w), 1020 (w), 936 (w), 880 (w), 836 (vs), 749 (w). Anal. Calcd for $C_{33}H_{53}SiPU$: C, 53.07; H, 7.15. Found: C, 52.91; H, 7.04.

Synthesis of $(C_5Me_5)_2U(CH_3)[-N=P(SiMe_3)_2(C_6H_5)]$, 3. To a scintillation vial charged with **1** (148 mg, 0.21 mmol), diethyl ether (5 mL), and a stir bar, azidotrimethylsilane (38 mg, 0.33 mmol) was added. Gas evolution was observed immediately. The solution was allowed to stir at room

temperature overnight, after which the solvent was removed under vacuum to yield a dark yellow-brown powder. Recrystallization from a concentrated diethyl ether solution (-40 °C) yielded dark yellow crystals which were dried *in vacuo* (30 mg, 18%). X-ray quality crystals were obtained from a concentrated diethyl ether solution at -40 °C. ^{1}H NMR (C_6D_6, 500 MHz, 298 K): δ 23.7 (s, 2H, *o*-Ph, $\nu_{1/2}$ = 18 Hz), 11.0 (s, 18H, Si*Me*$_3$, $\nu_{1/2}$ = 5 Hz), 9.12-9.09 (m, 1H, *p*-Ph), 8.88-8.87 (m, 2H, *m*-Ph), -1.83 (s, 30H, $C_5(CH_3)_5$, $\nu_{1/2}$ = 2 Hz), -194.4 (s, 3H, C*H*$_3$, $\nu_{1/2}$ = 85 Hz). ^{31}P{^{1}H} NMR (C_6D_6, 120 MHz): δ 518.0. IR (KBr, cm^{-1}): 2953 (m), 2901 (s), 2854 (m), 1436 (w), 1376 (w), 1246 (m), 1088 (m), 1046 (vs), 1023 (m), 933 (m), 882 (m), 839 (vs), 749 (w), 697 (w). Anal. Calcd for $C_{33}H_{56}NPSi_2U$: C, 50.05; H, 7.13; N, 1.77. Found: C, 49.86; H, 6.91; N, 1.72.

Synthesis of $(C_5Me_5)_2U(\kappa^2$-*N,N′*)–$[N(SiMe_3)]_2P(Mes)$, 4. To a scintillation vial charged with **2** (250 mg, 0.33 mmol), 1,2-dimethoxyethane (7 mL), and a stir bar, azidotrimethylsilane (73 mg, 0.63 mmol) was added. Gas evolution was observed immediately. The solution was let to stir at room temperature overnight, after which the solvent was removed *in vacuo* to yield a dark red-brown solid. Recrystallization from a concentrated diethyl ether solution (-40 °C) yielded dark red crystals which were dried *in vacuo* (175 mg, 66%). X-ray quality crystals were obtained over several days from a concentrated diethyl ether solution at -40 °C. ^{1}H NMR (C_6D_6, 500 MHz, 298 K): 33.7 (s, 3H, C*H*$_3$-Mes, $\nu_{1/2}$ = 15 Hz), 12.0 (s, 1H, C*H*-Mes, $\nu_{1/2}$ = 4 Hz), 10.7 (s, 15H, C_5Me_5, , $\nu_{1/2}$ = 5 Hz), 7.98 (s, 1H, C*H*-Mes, , $\nu_{1/2}$ = 7 Hz), 5.66 (s, 3H, C*H*$_3$-Mes, $\nu_{1/2}$ = 10 Hz), 5.01 (s, 15H, C_5Me_5, $\nu_{1/2}$ = 4 Hz), 3.70 (s, 3H, C*H*$_3$-Mes, $\nu_{1/2}$ = 4 Hz), -21.2 (s, 18H, Si*Me*$_3$, $\nu_{1/2}$ = 29 Hz). ^{31}P{^{1}H} NMR (C_6D_6, 120 MHz): δ -180.8. IR (KBr, cm^{-1}): 2949 (m), 2904 (br-m), 2856 (w), 1437 (br-w), 1376 (w), 1244 (s), 1083 (br-w), 1021 (br-w), 954 (w), 891 (s), 837 (vs), 765 (w), 689 (w), 668 (m). Anal. Calcd for $C_{35}H_{59}N_2PSi_2U$: C, 50.48; H, 7.14; N, 3.48. Found: C, 50.30; H, 7.27; N, 3.17.

Synthesis of $(C_5Me_5)_2U\{=N[P(SiMe_3)(Mes)]\}[=N(SiMe_3)]$, 5. To a 20 mL scintillation vial, 144 mg (0.193 mmol) of crystalline **3** was added and dissolved in ca. 5 mL of 1,2-dimethoxyethane. The solution was cooled to -45 °C and 2.5 mL of a 178 mM (0.443 mmol) Me_3SiN_3 in *n*-pentane solution was added dropwise over the course of one minute. The reaction mixture darkened instantly, and the reaction was allowed to warm for 5 minutes, after which volatiles are removed under reduced pressure. The solution was recrystallized from diethyl ether at -45 °C yielding 7.7 mg (0.0091 mmol) of analytically pure crystalline **5**. Further crystallizations gave greater yields, but with less pure product. X-ray quality crystals were grown from a saturated solution of diethyl ether at -45 °C. ^{1}H NMR (600 MHz, 25 °C, benzene-d_6): δ 1.03 9H (s, $SiMe_3$), 1.17 9H (s, $SiMe_3$), 3.14 3H (s, p-Me), 4.06 6H (s, o-Me), 4.67 30H (s, C_5Me_5), 8.13 2H (s, m C-H). ^{13}C{^{1}H} (600 MHz, 25 °C, benzene-d_6): δ 7.80 ($C_5\mathit{Me}_5$), 9.11 ($Si\mathit{Me}_3$), 13.77 (p-Me), 20.47 ($Si\mathit{Me}_3$), 32.72 (o-Me), 132.66 (m-CH), 137.47 (C_5Me_5), 140.72 (p-C), 165.31 (o-C), 178.04 (P-C). ^{31}P{^{1}H} (300 MHz, 25 °C, benzene-d_6): δ 157. IR (KBr, cm^{-1}): 2957 (w), 2899 (w), 2853 (w), 1603 (wb), 1436 (w), 1374 (w), 1260 (s), 1089 (vs), 1020 (s), 953 (s), 830 (s), 802 (s), 468 (w). Since **4** and **5** are structural isomers, their elemental analyses cannot be distinguished.

Synthesis of 4 from 5. 7.7 mg of **5** was dissolved in benzene-d_6 and 1,2-dimethoxyethane (ca. 2 mL) was added to a 20 mL scintillation vial. No immediate reaction was observed, but, after 48 h, volatiles were removed and the now red powder was dissolved in benzene-d_6, which showed conversion to **4**.

Crystallographic Data Collection and Structure Determination. The selected single crystal was coated with viscous hydrocarbon oil inside the glove box before being mounted on a nylon cryoloop using Parabar® hydrocarbon oil. The X-ray data were collected on a Bruker D8 Phaser diffractometer equipped with a Photon 100 CMOS area detector using Mo-Kα radiation (λ =

0.71073 Å) from a microfocus source. The data collection and processing utilized Bruker Apex3 suite of programs.[5] The structures were solved using an iterative dual space method as implemented in SHELXT and refined by full-matrix least-squares methods on F^2 using Bruker SHELX-2017/1 program.[6] All non-hydrogen atoms were refined with anisotropic displacement parameters. Hydrogen atoms were placed at calculated positions and included in the refinement using a riding model. Thermal ellipsoid plots were prepared by using Olex2[7] with 50% of probability displacements for non-hydrogen atoms.

The structure of **5** appears to be affected by twinning related to the presence of a pseudo-monoclinic supercell related by a transformation represented with the following 3x3 matrix: [(1 0 0) (2 2 0) (2 2 1)]. An attempt to solve the structure using the supercell as the unit cell resulted in an uninterpretable model. No twin matrix could be found, and introducing twin laws based for 2-fold rotation about *a* or reflection perpendicular to *a* (which is parallel to the special axis of the monoclinic supercell) failed to improve the model. The major consequence of this twinning is that atom C32 in the phenyl ring cannot be refined anisotropically. Attempting an anisotropic refinement of this atom inevitably results in it shifting severely out of the ring to occupy the position of a nearby major electron density difference peak. Modeling this peak as a uranium atom at 10% occupancy (and reducing the occupancy of U1 to 90%) allows C32 to be refined anisotropically but creates a number of other problems including highly unrealistic thermal ellipsoids for both U atoms and serious difference map peaks and holes. Separating the refinement of C32 from other parameters via the BLOC instruction does not improve the refinement. Ultimately, the best interpretation of this data set was found to be the presented model with all non-hydrogen atoms except C32 refined anisotropically with restraints added to all light atoms to enforce chemically reasonable thermal motion. This results in a model with no geometric restraints

that is chemically reasonable and fully consistent all other experimental results, particularly the conversion between its structurally characterized isomer **4**. We further note that any substitution or elimination at the position of C32 is incredibly unlikely as it would require breaking an aromatic ring.

Table S1. Crystallographic parameters for complexes **3**-**5**.

	**3**	**4**	**5**
**CCDC deposit number**	1826643	1826642	1995730
**Empirical formula**	$C_{33}H_{56}NPSi_2U$	$C_{35}H_{59}N_2PSi_2U$	$C_{35}H_{59}N_2PSi_2U$
**Formula weight (g/mol)**	791.96	833.02	833.02
**Crystal habit, color**	Prism, Dark Yellow	Prism, Dark Red	Prism, Black
**Temperature (K)**	100(2)	100(2)	100(2)
**Space group**	*P*-1	*C*c	*P*-1
**Crystal system**	Triclinic	Monoclinic	Triclinic
**Volume (Å^3)**	3573.6(3)	3661.7(4)	1849.0(4)
**a (Å)**	10.6409(6)	20.0922(13)	9.8231(11)
**b (Å)**	16.5027(9)	11.4805(6)	11.2078(13)
***c* (Å)**	20.4856(11)	18.3590(11)	19.039(3)
***α* (°)**	85.363(2)	90	82.061(4)
***β* (°)**	89.696(2)	120.155(3)	76.900(6)
***γ* (°)**	85.317(2)	90	65.055(4)

**Z**	4	4	2
**Calculated density (Mg/m^3)**	1.472	1.511	1.494
**Absorption coefficient (mm^{-1})**	4.675	4.567	4.522
**Final R indices [$I > 2\sigma(I)$]**	R_1 = 0.0391, wR_2 = 0.0667	R_1 = 0.0224, wR_2 = 0.0392	R_1 = 0.0715, wR_2 = 0.2236

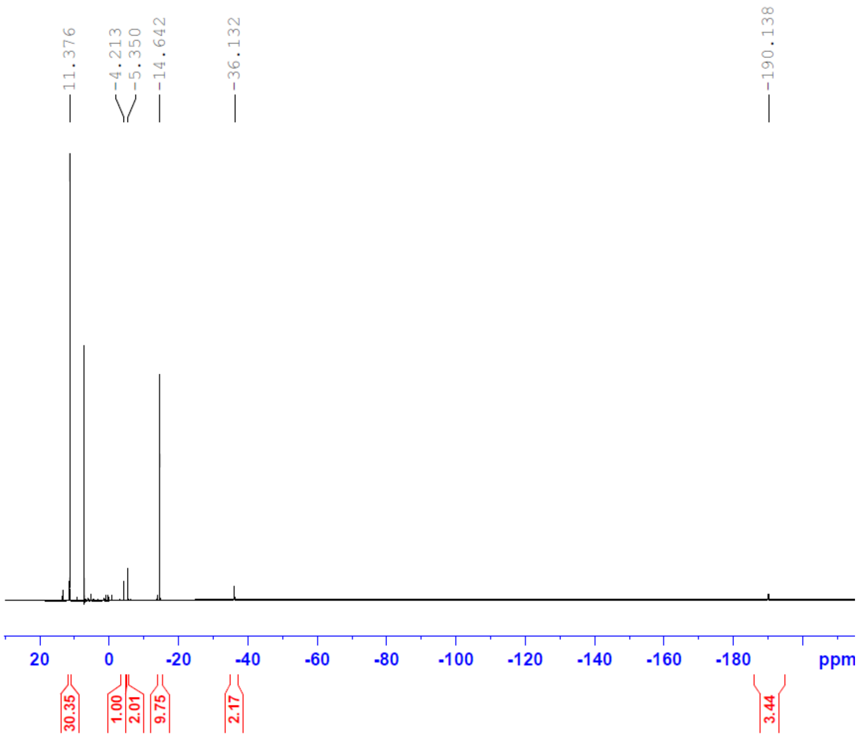

Figure S1. ^{1}H NMR spectrum of **1** in C_6D_6.

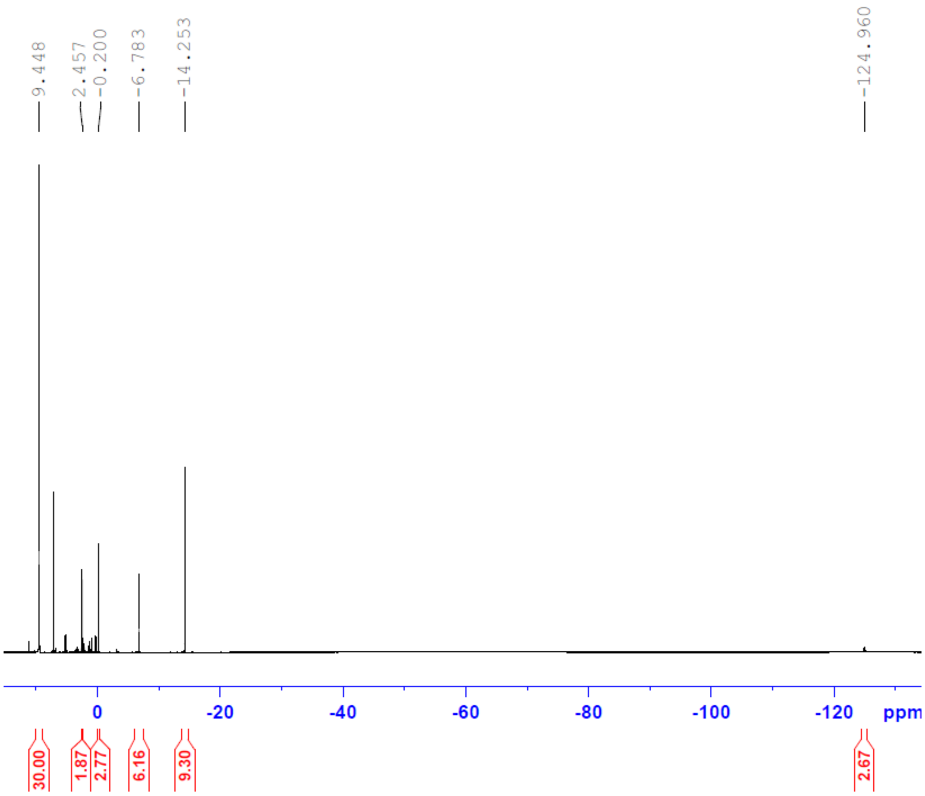

Figure S2. ^{1}H NMR spectrum of **2** in C_6D_6.

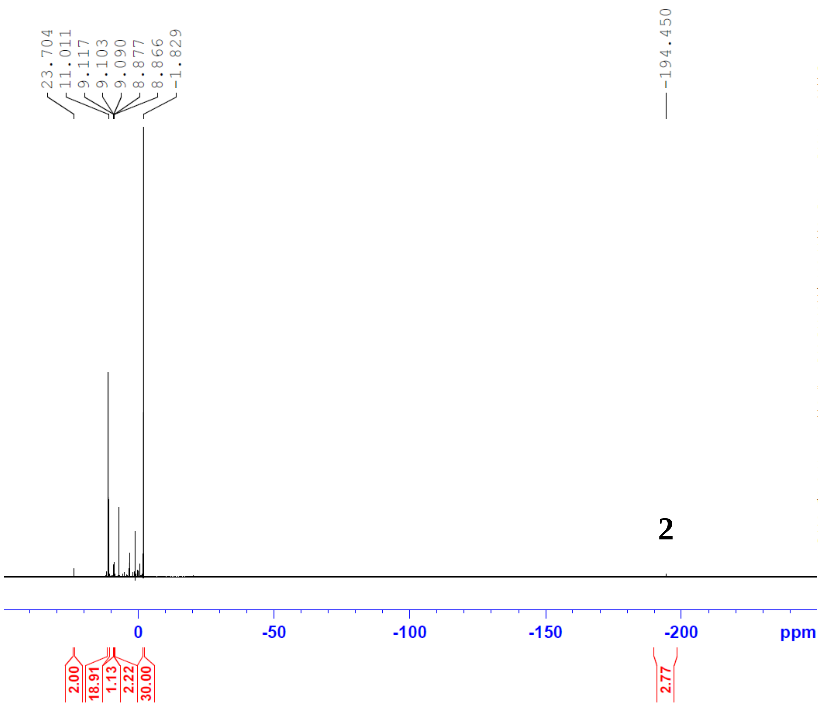

Figure S3. ^{1}H NMR spectrum of **3** in C_6D_6.

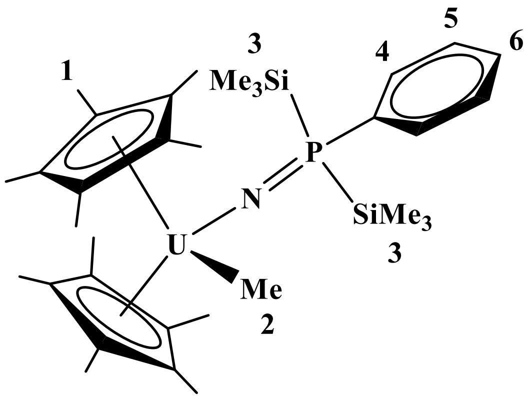

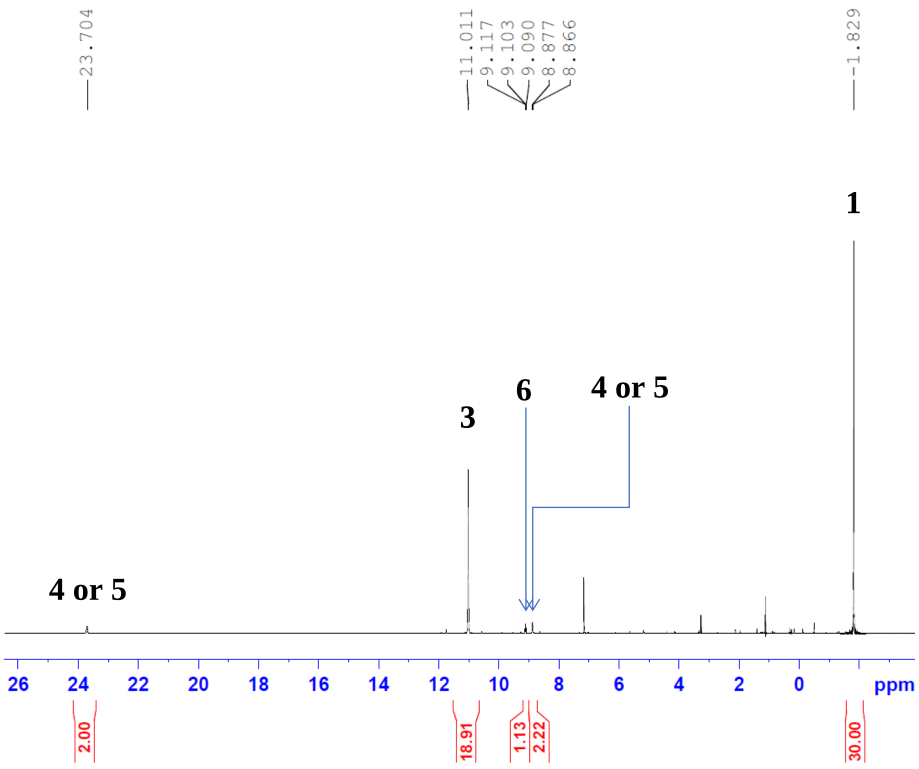

Figure S4. Close-up of the ^{1}H NMR spectrum of **3** in C_6D_6.

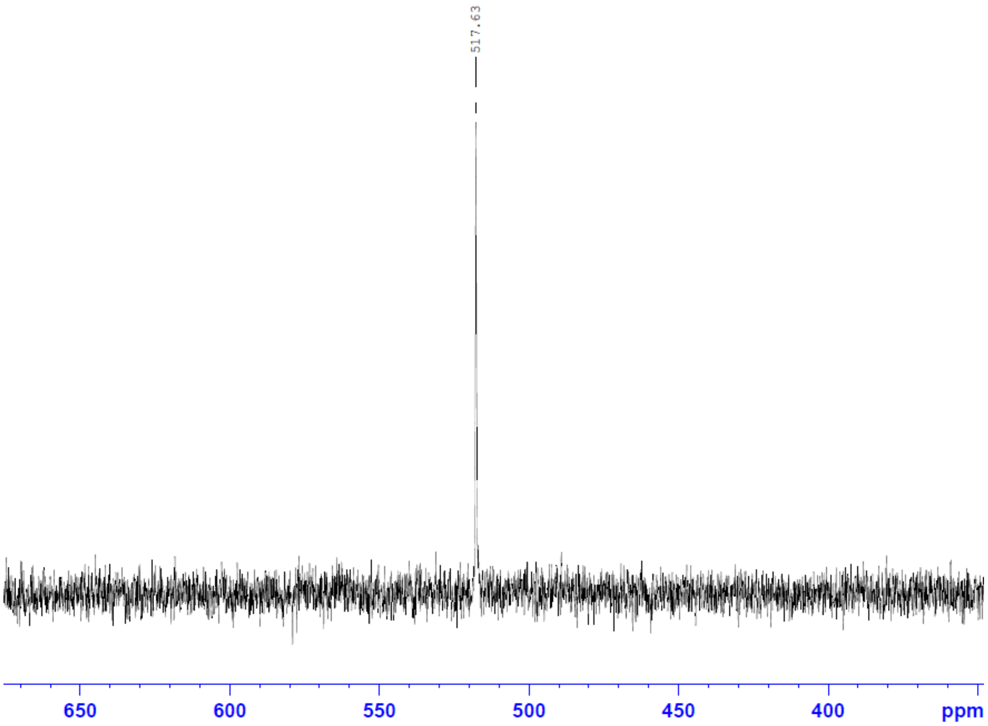

Figure S5. $^{31}P\{^{1}H\}$ NMR spectrum of **3** in C_6D_6.

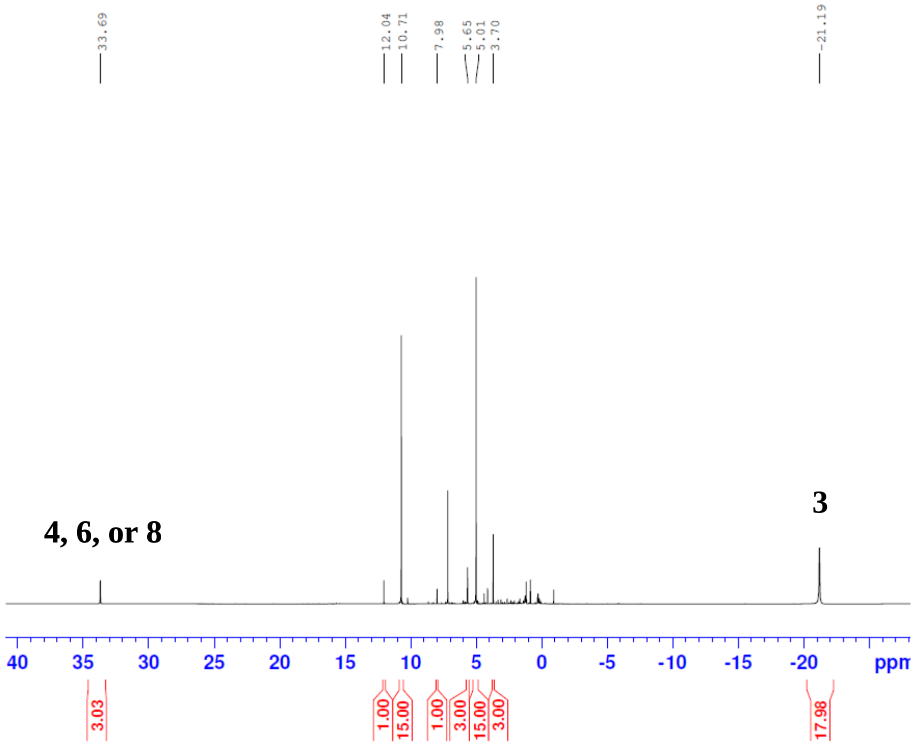

Figure S6. ^{1}H NMR spectrum of **4** in C_6D_6.

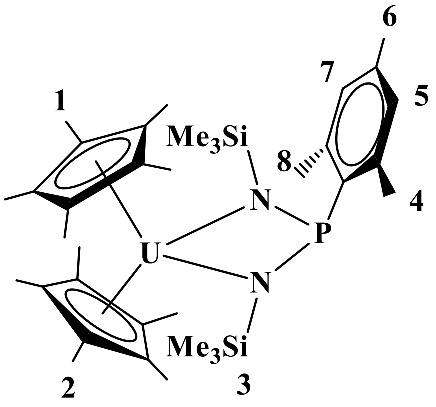

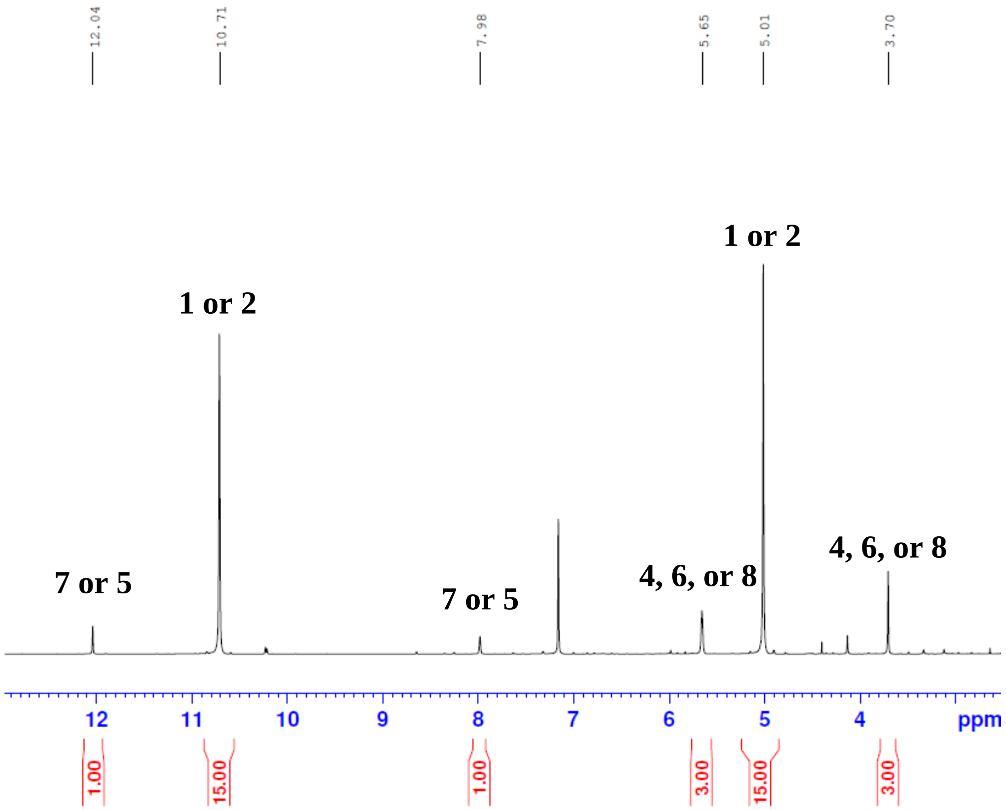

Figure S7. Close-up of the ^{1}H NMR spectrum of **4** in C_6D_6.

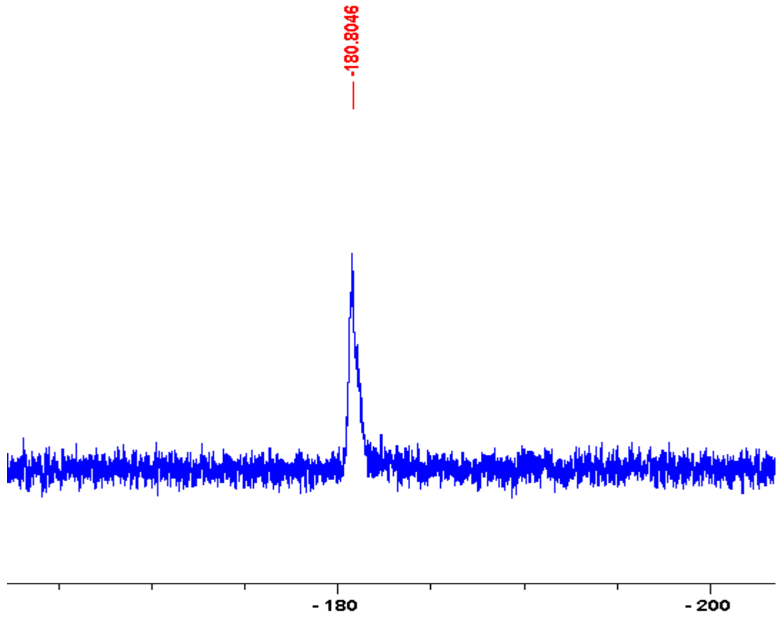

Figure S8. ^{31}P NMR spectrum of **4** in C_6D_6.

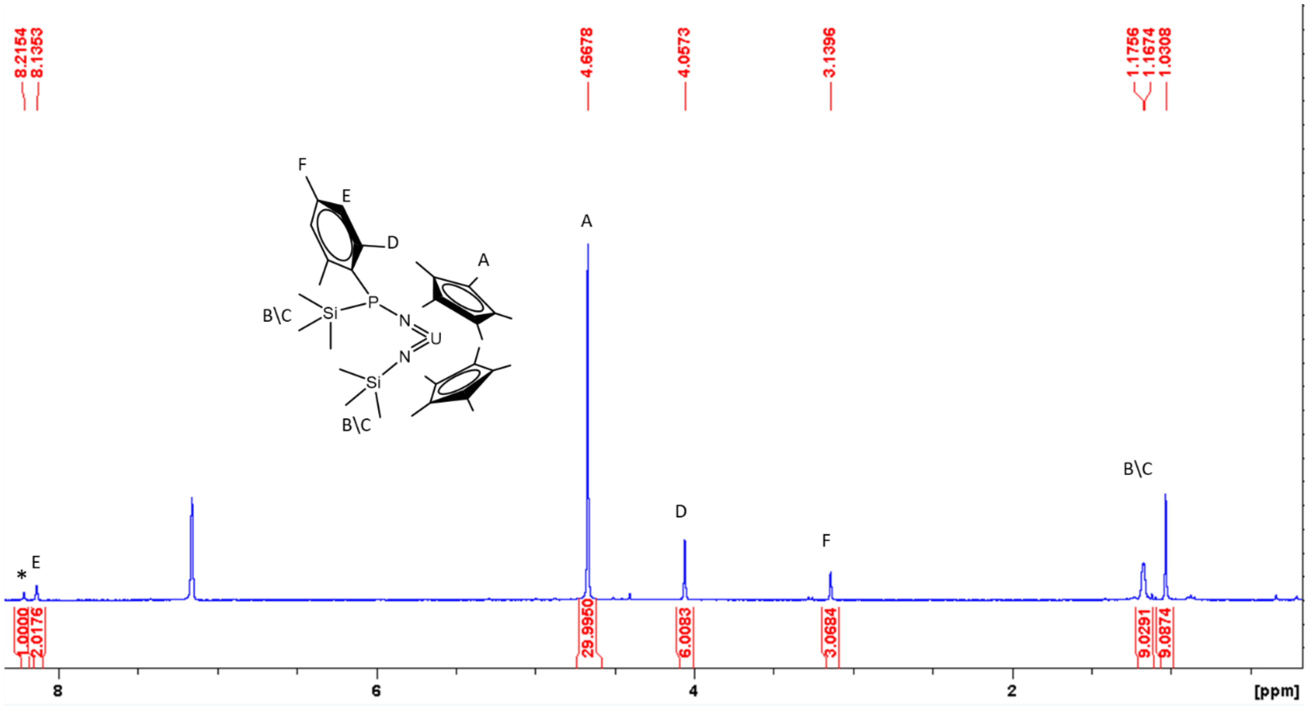

Figure S9. ^{1}H NMR spectrum of **5** in C_6D_6.

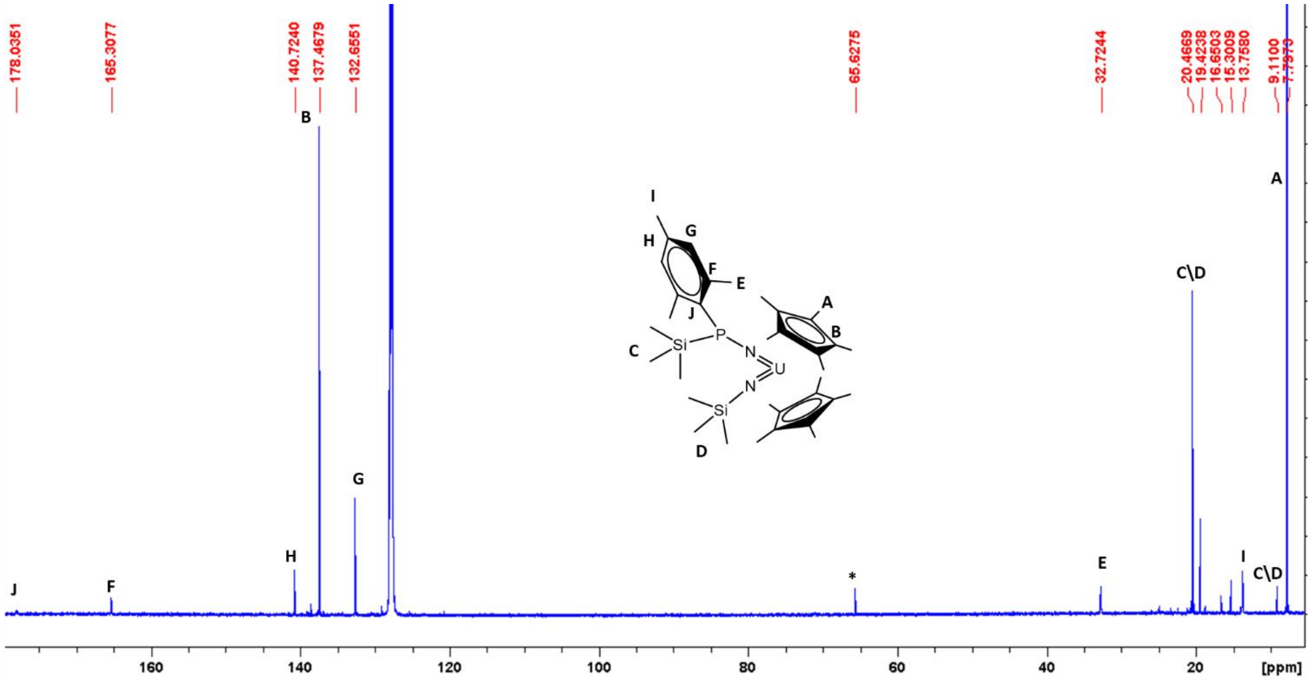

Figure S10. ^{13}C NMR spectrum of **5** in C_6D_6.

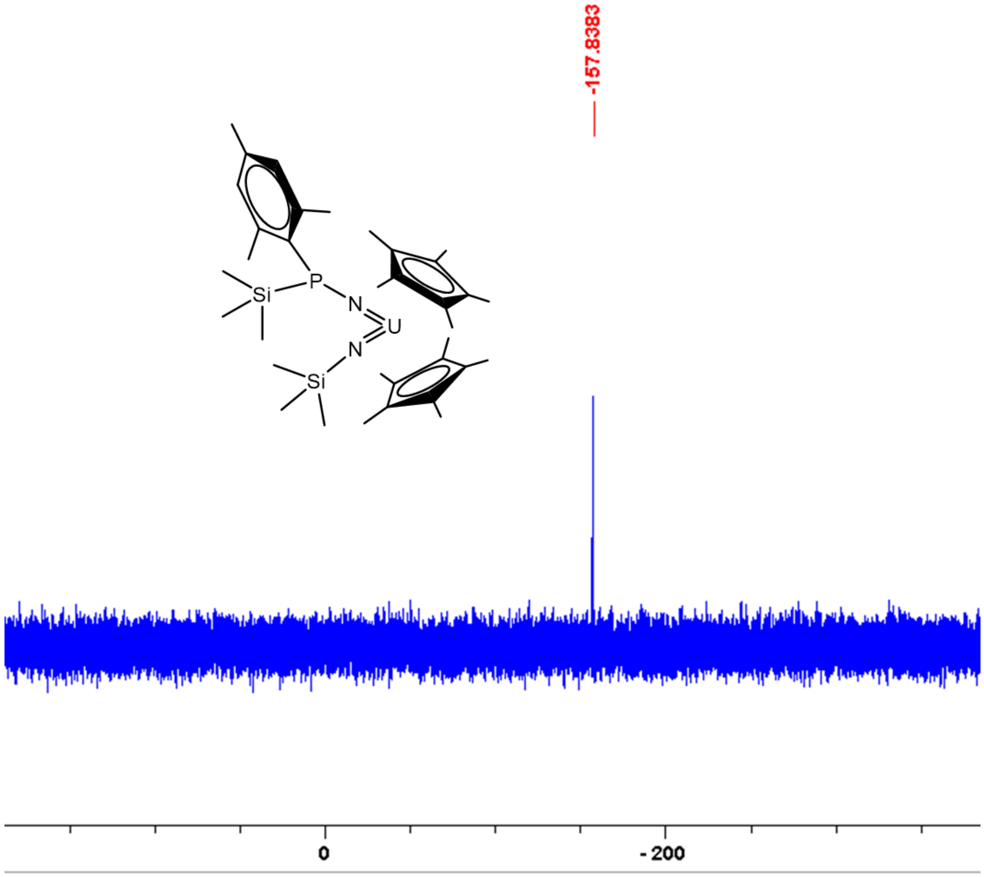

Figure S11. ^{31}P NMR spectrum of **5** in C_6D_6.

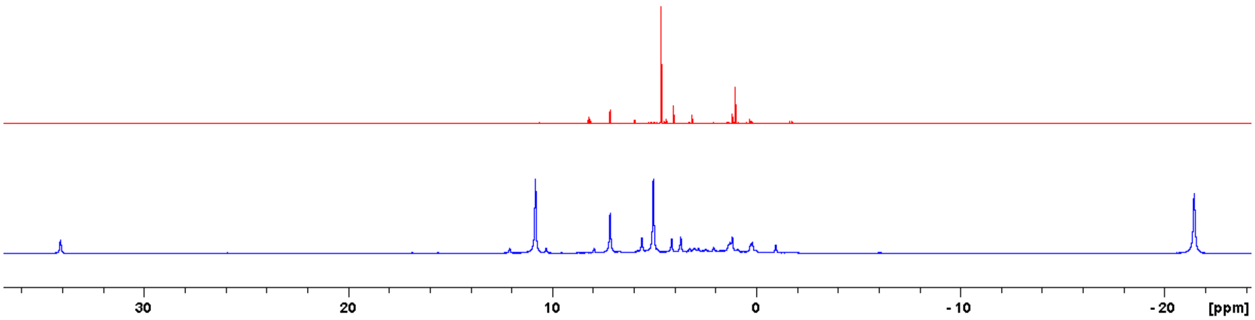

Figure S12. NMR spectra of the conversion of **5** (red) to **4** (blue).

Computational details. All DFT calculations were carried out with the Gaussian 09 suite of programs.[8] Geometries were fully optimized in gas phase without symmetry constraints, employing the B3PW91 functional.[9] The nature of the extrema was verified by analytical frequency calculations. The calculation of electronic energies and enthalpies of the extrema of the potential energy surface (minima and transition states) were performed at the same level of theory as the geometry optimizations. IRC calculations were performed to confirm the connections of the optimized transition states. The effect of a possible assistance of a solvent molecule (DME) during the formation of complex **4** from complex **5** was considered. However, no difference is observed with or without an explicit solvent molecule in interaction with the silicon atom of the $SiMe_3$ group. The abstraction of this same group by the solvent has also been envisaged. In this case, both from a kinetic and thermodynamic point of view, the reaction is not possible. Uranium atoms were treated with a small core effective core potential (60 MWB), associated with its adapted basis set.[10] Stuttgart effective core potentials[11] and their associated basis set were used for silicon augmented by a set of polarization functions ($\zeta d = 0.284$). For the other elements (H, C, N and P), Pople's double-ζ basis set 6-31G(d,p) was used.[12] The electronic charges (at the DFT level) were computed using the natural population analysis (NPA) technique.[13]

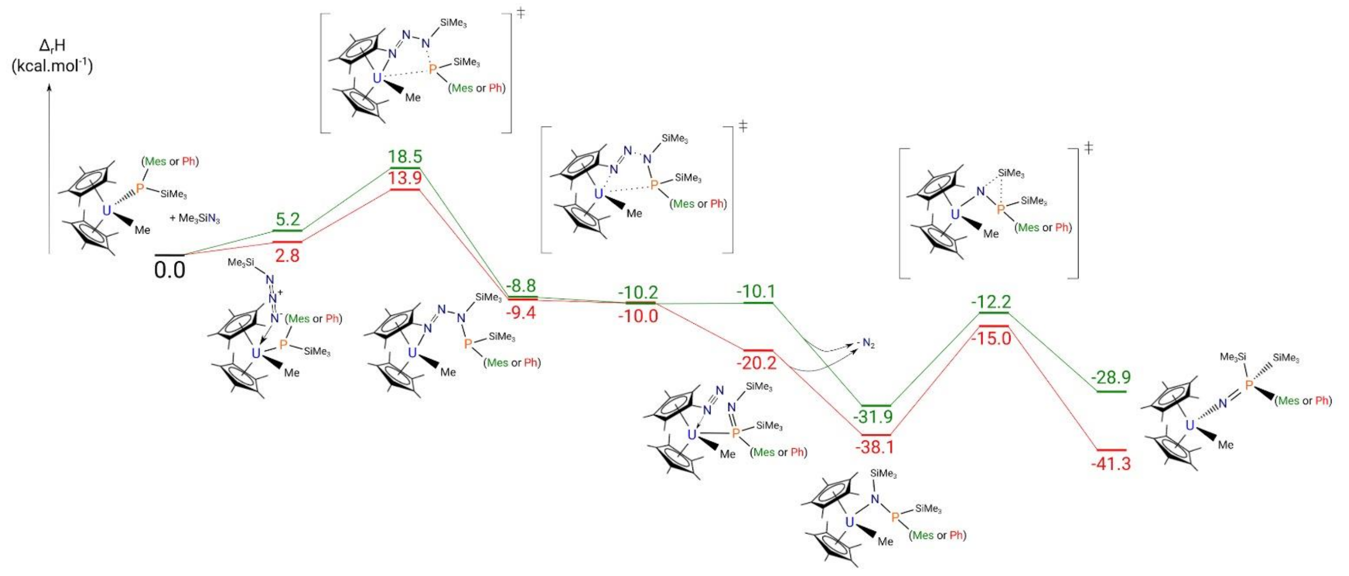

Figure S13. Computed enthalpy profile for the conversion of **1** into **3** and of **2** to **3**$^{\mathbf{Mes}}$

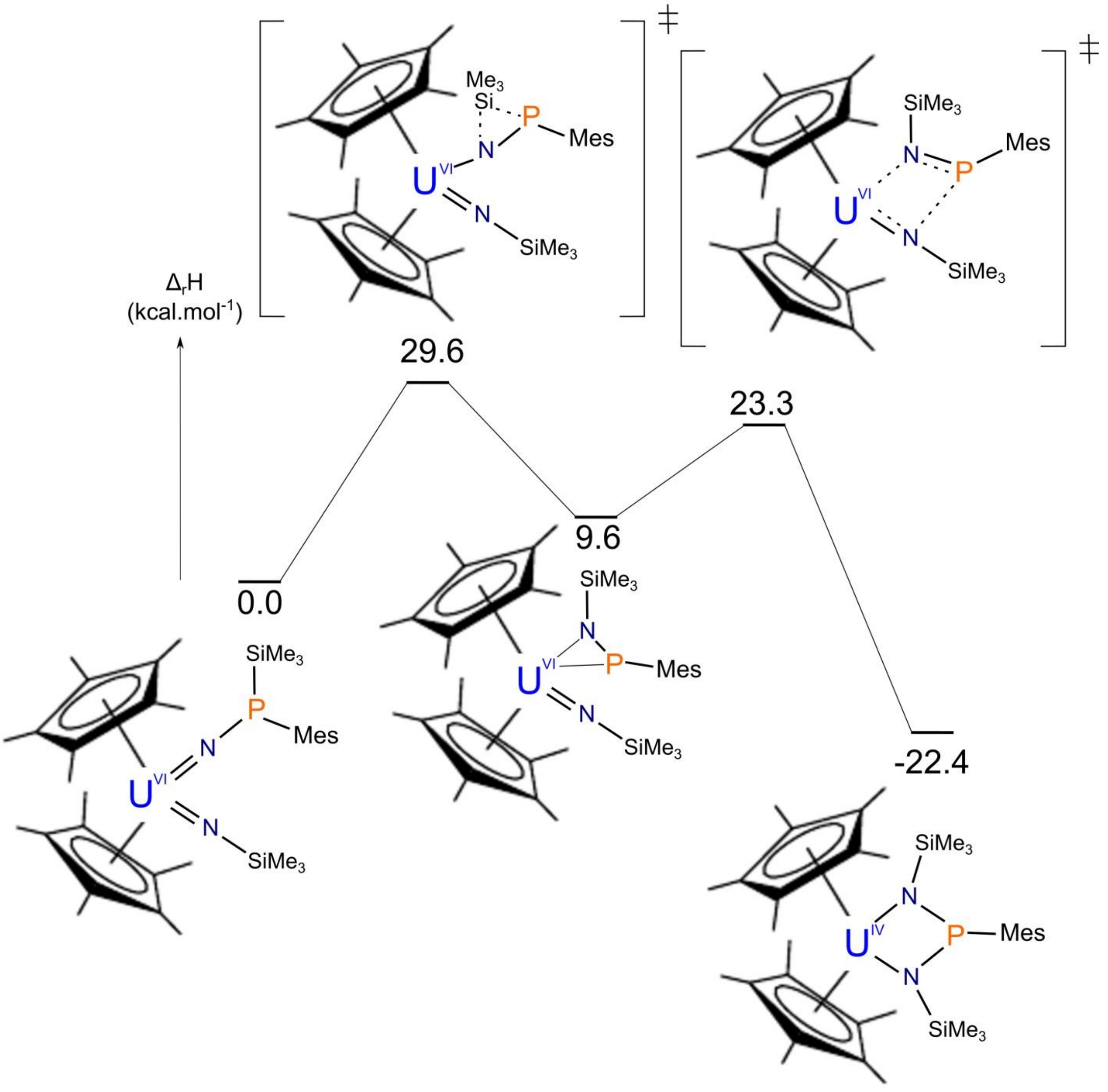

Figure S14. Computed enthalpy profile for the conversion of **5** into **4** on the singlet spin state potential energy surface.

Coordinates for calculated structures.

```
100
Complex 5
C        5.24914       7.84869       6.75090
C        4.33689       8.72294       7.34668
C        4.53249       9.23984       8.62758
C        5.68256       8.85210       9.31241
C        6.62926       7.98647       8.75459
C        6.41765       7.46775       7.45234
P        7.72220       6.33701       6.76666
N        8.00627       6.67224       5.12535
U        8.80067       7.41845       3.48639
N        7.95361       9.14527       3.37522
Si       7.15770      10.72487       3.51531
C        5.29830      10.63515       3.13566
C        7.83449       7.63739       9.59371
C        3.54912      10.20396       9.23547
C        4.95707       7.34232       5.36857
Si       6.83532       4.24166       7.11233
C        5.08652       3.82252       6.49531
C        7.33031      11.38821       5.28311
C        7.89696      11.97350       2.28872
C        8.08797       2.99865       6.41182
C        6.80461       4.06597       9.00469
C        6.91689       6.72110       1.58964
C        7.27155       5.48551       2.19927
C        8.59337       5.15225       1.78663
C        9.04398       6.17162       0.89829
C        8.02163       7.15708       0.80230
C       10.23207       6.09223      -0.01198
C        9.27737       3.84881       2.07921
C        6.34018       4.57006       2.93066
C        5.54761       7.32731       1.57699
C        8.03730       8.34731      -0.10974
C       10.76324       8.31284       5.19611
C       11.12640       6.95010       4.98181
C       11.58426       6.82330       3.64200
C       11.50241       8.10895       3.02435
C       11.01053       9.02842       3.99120
C       12.28297       5.61307       3.09852
C       11.21612       5.88593       6.03199
C       12.06591       8.51123       1.69270
C       10.38500       8.91577       6.51425
C       11.00172      10.51576       3.81974
H       10.45679       6.01857       6.80766
H       12.20117       5.90519       6.51896
H       11.08649       4.88421       5.61047
H       11.81043       4.67941       3.41622
H       13.32010       5.58043       3.45941
```

H	12.32680	5.61057	2.00738
H	10.30849	3.84221	1.71927
H	8.75790	3.01495	1.58851
H	9.30145	3.61754	3.15033
H	10.75398	10.80597	2.79579
H	11.99344	10.93236	4.04578
H	10.28671	11.00133	4.48638
H	6.85645	3.98266	3.69308
H	5.87133	3.86073	2.23400
H	5.53712	5.12229	3.42269
H	11.28157	9.17487	7.09400
H	9.79413	8.21803	7.11322
H	9.79736	9.82885	6.39387
H	10.62948	7.07722	-0.26747
H	9.94273	5.61293	-0.95742
H	11.04747	5.49705	0.40526
H	12.37476	7.64707	1.10194
H	12.95317	9.14400	1.82735
H	11.35768	9.09150	1.08980
H	7.36806	9.13357	0.24738
H	7.71706	8.07609	-1.12522
H	9.03670	8.78527	-0.19712
H	5.00986	7.16494	2.51394
H	4.94158	6.88319	0.77520
H	5.58451	8.40320	1.40247
H	6.93346	10.66731	6.00425
H	6.76799	12.32281	5.39216
H	8.37117	11.59658	5.54720
H	8.93276	12.22647	2.52918
H	7.31294	12.90104	2.30613
H	7.87105	11.58316	1.26628
H	5.09860	10.44736	2.07651
H	4.84125	11.59935	3.38817
H	4.79509	9.86312	3.72315
H	8.17467	3.07531	5.32453
H	7.78478	1.97418	6.65475
H	9.07871	3.17219	6.84251
H	7.77559	6.61733	9.98792
H	7.91347	8.31498	10.44893
H	8.76207	7.69456	9.01801
H	7.80133	4.19103	9.43771
H	6.44239	3.06728	9.27452
H	6.13265	4.79931	9.46086
H	5.85805	9.23858	10.31495
H	4.35220	4.53692	6.87913
H	4.81216	2.82825	6.86834
H	5.01480	3.80247	5.40551
H	3.76657	11.23468	8.92861
H	3.58259	10.17569	10.32861
H	2.52437	9.98275	8.92097
H	3.93238	7.58422	5.07059
H	5.08920	6.25918	5.30896

H	5.65027	7.77911	4.64348
H	3.44196	9.00095	6.79232

100

U(IV) TS P-Si activation

C	-0.45614	-1.27482	2.64451
C	-0.79721	0.06964	2.96336
C	-2.17158	0.26739	2.65110
C	-2.68715	-0.97009	2.16118
C	-1.62527	-1.91401	2.14292
C	0.06639	1.00397	3.75620
C	-2.96359	1.49594	2.99120
C	-4.12980	-1.34714	2.00101
C	-1.79311	-3.37730	1.86773
C	0.82376	-1.95209	3.02962
U	-0.86556	-0.13402	0.14891
N	-0.04781	-1.72787	-0.56274
Si	0.69561	-3.20851	-1.17898
C	-0.63099	-4.46511	-1.70930
N	0.97575	1.06262	0.24699
Si	1.96103	2.97915	0.96597
C	2.98271	3.51346	2.49280
P	2.43800	0.64756	0.94718
C	3.82171	0.23036	-0.19794
C	3.80081	0.20411	-1.61434
C	4.95790	-0.15541	-2.31054
C	6.14806	-0.48664	-1.66342
C	6.15679	-0.46021	-0.26739
C	5.02771	-0.11734	0.47496
C	2.57585	0.55806	-2.40845
C	7.38718	-0.84067	-2.43980
C	5.14105	-0.14277	1.98229
C	1.81513	-4.05136	0.10600
C	1.80921	-2.90431	-2.68662
C	0.27849	3.81094	1.20277
C	2.79396	3.70587	-0.57040
C	-2.54990	1.83097	-1.13955
C	-1.52274	1.48508	-2.06320
C	-1.73059	0.13490	-2.46491
C	-2.88464	-0.35471	-1.78439
C	-3.40610	0.70549	-0.99183
C	-0.56054	2.44812	-2.69167
C	-2.87276	3.20696	-0.63328
C	-4.78818	0.78668	-0.41793
C	-3.53752	-1.68355	-2.03013
C	-1.02970	-0.56999	-3.58648
H	-0.12584	3.13993	-1.96557
H	-1.06679	3.05269	-3.45664
H	0.26340	1.92966	-3.18451
H	1.28982	-2.38464	-3.49554
H	2.15123	-3.87120	-3.07487
H	2.69503	-2.32344	-2.41510
H	-4.21591	-1.96177	-1.21841

H	-2.80139	-2.48536	-2.12942
H	-4.13287	-1.66927	-2.95335
H	-5.23615	-0.19477	-0.25700
H	-5.44086	1.32314	-1.12136
H	-4.82901	1.33865	0.52535
H	1.00226	-2.85092	2.43843
H	0.79550	-2.25192	4.08674
H	1.68835	-1.29508	2.89807
H	3.73189	3.20018	-0.81327
H	3.02063	4.75841	-0.36155
H	2.14013	3.67021	-1.44482
H	2.61493	3.03915	3.40703
H	2.91136	4.60092	2.62088
H	4.03632	3.24662	2.36940
H	2.32396	1.61432	-2.28961
H	1.70546	-0.00981	-2.07405
H	2.74063	0.36372	-3.47222
H	-4.78943	-0.47882	2.01038
H	-4.43756	-1.99421	2.83357
H	-4.32528	-1.90830	1.08160
H	0.02317	-0.28823	-3.66235
H	-1.50222	-0.32670	-4.54840
H	-1.07177	-1.65468	-3.46724
H	-3.81712	3.56280	-1.06760
H	-2.10487	3.93229	-0.91192
H	-2.99275	3.25052	0.45484
H	-2.42255	2.41908	2.75676
H	-3.19802	1.53205	4.06386
H	-3.91581	1.53226	2.45611
H	-2.49708	-3.56466	1.05127
H	-2.18119	-3.89894	2.75407
H	-0.84627	-3.84853	1.59895
H	-0.34315	3.70134	0.31463
H	0.44203	4.87925	1.39061
H	-0.26151	3.39812	2.05704
H	4.92261	-0.17624	-3.39836
H	-1.18271	-4.12044	-2.58992
H	-1.35192	-4.65599	-0.90939
H	-0.15931	-5.41937	-1.97172
H	4.92891	0.83231	2.43345
H	6.15284	-0.43240	2.27971
H	4.44179	-0.85599	2.43222
H	2.37770	-4.85535	-0.38342
H	1.26009	-4.50095	0.93485
H	2.53961	-3.34310	0.51907
H	1.11674	0.95540	3.45498
H	0.02589	0.74038	4.82246
H	-0.26333	2.04281	3.67817
H	7.07025	-0.72387	0.26301
H	7.95522	-1.63570	-1.94649
H	8.05532	0.02494	-2.53109
H	7.14405	-1.17435	-3.45256

100
U(IV) intermediate

C	-0.53381	-1.25608	2.67448
C	-0.83563	0.10386	2.95088
C	-2.20117	0.33328	2.61222
C	-2.74827	-0.89826	2.14204
C	-1.70988	-1.86758	2.15437
C	0.05614	1.07273	3.66555
C	-2.96065	1.58659	2.93381
C	-4.19935	-1.26034	2.02732
C	-1.90934	-3.33300	1.91349
C	0.70131	-1.99538	3.09152
U	-0.94225	-0.11254	0.13313
N	-0.31549	-1.75502	-0.68388
Si	0.42727	-3.23837	-1.30805
C	-0.90757	-4.49285	-1.81432
N	0.89957	1.47119	0.32611
Si	1.54172	3.13279	0.50892
C	2.80496	3.19688	1.92459
P	1.85351	0.15339	0.72291
C	3.45665	0.11334	-0.19680
C	3.58191	0.28884	-1.59515
C	4.84131	0.18036	-2.18997
C	5.99043	-0.10392	-1.45093
C	5.84690	-0.30946	-0.07817
C	4.60908	-0.21611	0.56113
C	2.39215	0.55999	-2.47199
C	7.34057	-0.17740	-2.11136
C	4.55090	-0.48276	2.04699
C	1.55514	-4.05265	-0.01516
C	1.53761	-2.93852	-2.81942
C	0.14279	4.33142	0.94083
C	2.46048	3.81124	-1.00835
C	-2.65353	1.85478	-1.08985
C	-1.65379	1.53142	-2.05198
C	-1.86426	0.18971	-2.46992
C	-2.99402	-0.31951	-1.76142
C	-3.49860	0.72122	-0.93595
C	-0.73909	2.52011	-2.70899
C	-2.97764	3.22409	-0.56679
C	-4.88475	0.79658	-0.36922
C	-3.65779	-1.63976	-2.01783
C	-1.20023	-0.48669	-3.62997
H	-0.31982	3.23463	-1.99731
H	-1.28249	3.09777	-3.46966
H	0.09381	2.02966	-3.21597
H	1.01975	-2.42652	-3.63409
H	1.88361	-3.90720	-3.19955
H	2.42266	-2.35468	-2.55074
H	-4.25913	-1.96797	-1.16497
H	-2.92797	-2.42591	-2.22221
H	-4.33298	-1.58215	-2.88284

H	-5.33585	-0.18625	-0.22805
H	-5.52921	1.34355	-1.07243
H	-4.93620	1.33460	0.58090
H	1.07441	-2.65328	2.30391
H	0.49148	-2.61691	3.97290
H	1.50827	-1.30959	3.35722
H	3.33911	3.20279	-1.24197
H	2.81251	4.82195	-0.76891
H	1.83944	3.88325	-1.90483
H	2.38705	2.84392	2.87132
H	3.13493	4.23236	2.06815
H	3.69072	2.59840	1.69069
H	2.05953	1.59530	-2.37797
H	1.54575	-0.07651	-2.19688
H	2.63538	0.37412	-3.52211
H	-4.84912	-0.38538	2.04781
H	-4.48651	-1.89381	2.87766
H	-4.42917	-1.83029	1.12170
H	-0.15155	-0.19886	-3.73757
H	-1.70632	-0.22348	-4.56925
H	-1.23544	-1.57384	-3.53389
H	-4.01054	3.49418	-0.82260
H	-2.33193	3.98368	-1.01304
H	-2.89075	3.31500	0.52116
H	-2.38023	2.49067	2.72114
H	-3.22042	1.62525	4.00060
H	-3.89543	1.65812	2.37340
H	-2.59246	-3.52302	1.08052
H	-2.33883	-3.81897	2.80137
H	-0.96717	-3.83660	1.68966
H	-0.55023	4.48482	0.11147
H	0.57499	5.30525	1.19764
H	-0.43134	3.98430	1.80507
H	4.92126	0.31056	-3.26793
H	-1.46208	-4.15965	-2.69731
H	-1.62457	-4.66716	-1.00702
H	-0.44064	-5.45291	-2.06332
H	4.17623	0.38083	2.60685
H	5.54646	-0.72349	2.43078
H	3.89203	-1.32544	2.28326
H	2.10146	-4.88144	-0.48070
H	1.00781	-4.45825	0.84046
H	2.29311	-3.33392	0.35437
H	1.10855	0.91587	3.41247
H	-0.04132	0.96147	4.75420
H	-0.18869	2.11075	3.42449
H	6.72486	-0.55595	0.51633
H	8.01293	-0.85442	-1.57611
H	7.82114	0.80875	-2.13342
H	7.26228	-0.52292	-3.14658

100

U(IV) TS P - N bond formation

C	5.06907	7.35816	10.45275
C	4.70429	8.71035	10.69025
C	3.32880	8.86611	10.34610
C	2.84194	7.59883	9.91129
C	3.92940	6.68236	9.93652
C	5.55636	9.72948	11.38294
C	2.51371	10.09967	10.60755
C	1.40871	7.16566	9.82604
C	3.81601	5.20419	9.71158
C	6.32880	6.68916	10.91230
U	4.64271	8.45750	7.88146
N	5.80448	6.99708	7.16269
Si	6.63232	5.52905	6.61065
C	5.34329	4.18254	6.23101
N	6.46159	9.98691	8.02560
Si	6.98356	11.68191	8.19943
C	8.26914	11.83897	9.58764
P	7.44411	8.70384	8.43860
C	8.96858	8.64101	7.38775
C	9.06609	8.91809	6.00366
C	10.31650	8.88100	5.38197
C	11.48527	8.57505	6.08014
C	11.37333	8.27821	7.43880
C	10.14681	8.29879	8.10558
C	7.85872	9.21829	5.16459
C	12.82350	8.57635	5.39284
C	10.14842	7.96478	9.58188
C	7.81174	4.81989	7.91967
C	7.66559	5.74995	5.03323
C	5.49079	12.75929	8.63085
C	7.81046	12.43532	6.66562
C	2.84471	10.11868	6.41510
C	3.87177	9.75835	5.49629
C	3.74652	8.36681	5.22589
C	2.64779	7.86793	5.98275
C	2.07052	8.95986	6.69106
C	4.71370	10.73385	4.72788
C	2.45289	11.51207	6.81397
C	0.68667	9.01578	7.26544
C	2.07299	6.48597	5.86618
C	4.45633	7.60788	4.14709
H	5.13875	11.51739	5.36102
H	4.11013	11.23475	3.95846
H	5.54003	10.24138	4.21321
H	7.07955	6.16223	4.20774
H	8.03624	4.76652	4.71967
H	8.53318	6.39408	5.19730
H	1.43028	6.23751	6.71556
H	2.85170	5.72057	5.81043
H	1.45785	6.38724	4.96096
H	0.28480	8.02744	7.49141
H	0.00849	9.47523	6.53211

H	0.62068	9.62356	8.17242
H	6.75008	6.03027	10.15058
H	6.12916	6.08119	11.80552
H	7.09809	7.41795	11.17481
H	8.72161	11.89570	6.39092
H	8.09781	13.46602	6.90686
H	7.15296	12.47435	5.79261
H	7.88580	11.49594	10.55229
H	8.56157	12.88975	9.69647
H	9.17448	11.26914	9.35349
H	7.56696	10.26597	5.25439
H	7.00387	8.61579	5.48023
H	8.06503	9.01492	4.10949
H	0.71946	8.00791	9.76346
H	1.14525	6.60246	10.73208
H	1.20911	6.50209	8.97903
H	5.46123	7.99267	3.95999
H	3.90131	7.67449	3.20078
H	4.55112	6.54823	4.39385
H	2.39991	11.65539	7.89938
H	1.45941	11.76118	6.41701
H	3.14814	12.25465	6.41499
H	3.04385	11.01728	10.32832
H	2.26594	10.19385	11.67362
H	1.56853	10.09174	10.05905
H	3.15423	4.96185	8.87407
H	3.40551	4.70085	10.59876
H	4.78993	4.75431	9.50498
H	4.75963	12.78855	7.81977
H	5.82444	13.78698	8.81360
H	4.98391	12.40836	9.53426
H	10.37308	9.08394	4.31382
H	4.75451	4.41729	5.33895
H	4.65438	4.03024	7.06606
H	5.85791	3.23298	6.04147
H	9.87028	8.82690	10.19767
H	11.14760	7.64542	9.89173
H	9.44915	7.16078	9.83076
H	8.38173	3.99332	7.47943
H	7.27864	4.42591	8.78966
H	8.53067	5.56976	8.26155
H	6.60656	9.64319	11.08818
H	5.51021	9.60229	12.47320
H	5.23300	10.75092	11.16548
H	12.26855	8.01722	8.00026
H	13.28165	9.57258	5.43115
H	12.73256	8.30155	4.33780
H	13.51995	7.87985	5.86883

100

Complex 4

C	-0.82987	-1.36430	2.62994
C	-0.99268	0.01624	2.93318

C	-2.33359	0.38454	2.61289
C	-3.00167	-0.77671	2.13344
C	-2.06491	-1.84722	2.11180
C	0.00842	0.83816	3.68565
C	-2.98362	1.69959	2.93649
C	-4.48404	-0.99244	2.07141
C	-2.43741	-3.27871	1.85055
C	0.35628	-2.18680	3.02883
U	-1.06228	-0.05624	0.15987
N	0.81095	-1.23891	-0.24513
Si	1.39981	-2.84158	-0.73979
C	-0.04150	-4.07406	-0.76927
N	0.77175	1.22834	0.38082
Si	1.33759	2.88005	0.71331
C	2.69077	2.95760	2.04227
P	1.89692	-0.11552	0.56029
C	3.40915	0.16126	-0.53425
C	3.45003	0.44759	-1.91410
C	4.67897	0.58474	-2.56981
C	5.89081	0.45576	-1.89779
C	5.84550	0.16832	-0.53259
C	4.64289	0.01459	0.16208
C	2.20512	0.63481	-2.73148
C	7.20561	0.63599	-2.60796
C	4.74913	-0.32032	1.63648
C	2.73076	-3.53015	0.42426
C	2.16265	-2.84705	-2.47636
C	-0.10662	3.97378	1.27667
C	2.08406	3.71958	-0.81662
C	-2.67638	1.60556	-1.45576
C	-1.68172	1.10332	-2.34263
C	-1.90497	-0.29145	-2.50279
C	-3.06248	-0.64177	-1.74116
C	-3.55981	0.54079	-1.12946
C	-0.78180	1.97391	-3.16708
C	-2.94428	3.05551	-1.17172
C	-4.93166	0.76776	-0.56986
C	-3.73616	-1.98338	-1.77581
C	-1.22005	-1.19588	-3.48518
H	-0.14594	2.62270	-2.55941
H	-1.38524	2.62244	-3.81582
H	-0.13134	1.38664	-3.81610
H	1.45542	-2.51811	-3.24195
H	2.47053	-3.87035	-2.72263
H	3.04918	-2.20993	-2.52813
H	-4.47287	-2.09183	-0.97558
H	-3.02691	-2.81326	-1.68170
H	-4.26662	-2.13499	-2.72560
H	-5.44690	-0.16019	-0.32121
H	-5.54631	1.28453	-1.31994
H	-4.93005	1.40206	0.32155
H	0.36138	-3.16338	2.53962

H	0.34744	-2.36576	4.11292
H	1.29739	-1.68608	2.78061
H	2.94799	3.16375	-1.19152
H	2.42472	4.72525	-0.54332
H	1.36198	3.82573	-1.63134
H	2.41495	2.44381	2.96625
H	2.88402	4.00897	2.28648
H	3.62856	2.53144	1.67415
H	2.04029	1.69730	-2.93163
H	1.32648	0.24614	-2.21846
H	2.29980	0.13219	-3.70025
H	-5.04585	-0.05881	2.02771
H	-4.80980	-1.51608	2.98151
H	-4.79342	-1.61534	1.22741
H	-0.21025	-0.85460	-3.72257
H	-1.78170	-1.23743	-4.42877
H	-1.13922	-2.22373	-3.12051
H	-3.25665	3.23467	-0.13712
H	-3.75157	3.43630	-1.81344
H	-2.06542	3.67532	-1.36583
H	-2.30700	2.54638	2.79025
H	-3.31023	1.73226	3.98466
H	-3.87001	1.88099	2.32120
H	-2.79494	-3.45884	0.83032
H	-3.24418	-3.59279	2.52578
H	-1.59604	-3.95203	2.02635
H	-0.92231	3.98323	0.54899
H	0.24830	5.00559	1.38051
H	-0.50952	3.66803	2.24578
H	4.67973	0.79833	-3.63801
H	-0.90546	-3.69864	-1.32667
H	-0.37550	-4.33644	0.23701
H	0.28498	-4.99710	-1.26190
H	4.23829	0.40801	2.27114
H	5.80244	-0.34636	1.93246
H	4.31040	-1.29422	1.87167
H	2.94667	-4.56948	0.14993
H	2.41067	-3.51332	1.46850
H	3.66357	-2.96530	0.33898
H	1.02716	0.62371	3.35082
H	-0.04346	0.61656	4.76067
H	-0.16465	1.91053	3.57039
H	6.78067	0.05141	0.01247
H	7.61684	1.63657	-2.42579
H	7.09645	0.51857	-3.68989
H	7.95082	-0.08672	-2.26052

100
U(VI) TS P-Si activation

C	-0.34316	-1.27466	2.63294
C	-0.71730	0.05945	2.97220
C	-2.07682	0.24340	2.60888
C	-2.55525	-0.99238	2.06941

C	-1.47726	-1.92133	2.07646
C	0.09394	0.99473	3.81675
C	-2.89024	1.45702	2.95411
C	-3.98602	-1.41550	1.92108
C	-1.61280	-3.38675	1.80046
C	0.92612	-1.94931	3.05287
U	-0.67710	-0.07066	0.12340
N	0.03422	-1.69286	-0.63555
Si	0.80167	-3.17034	-1.27370
C	-0.52079	-4.44746	-1.75446
N	0.64629	1.49265	0.03313
Si	1.89650	3.00553	0.88232
C	3.32221	3.34625	2.13073
P	1.94134	0.61113	0.87170
C	3.42486	0.19817	-0.17528
C	3.55493	0.27532	-1.58119
C	4.78038	-0.03577	-2.17767
C	5.89634	-0.42866	-1.43894
C	5.74970	-0.52123	-0.05628
C	4.54711	-0.21930	0.58744
C	2.41282	0.66632	-2.47464
C	7.21223	-0.72000	-2.10863
C	4.49685	-0.37397	2.08954
C	1.96202	-3.95927	0.00385
C	1.87011	-2.84832	-2.80676
C	0.44289	3.98315	1.60361
C	2.54303	3.79149	-0.71580
C	-2.40563	1.81395	-1.19204
C	-1.46583	1.40323	-2.17113
C	-1.70712	0.03075	-2.46301
C	-2.82271	-0.39884	-1.68276
C	-3.27356	0.71629	-0.92301
C	-0.56698	2.33285	-2.92448
C	-2.62463	3.21995	-0.72193
C	-4.62648	0.88785	-0.30155
C	-3.51461	-1.72059	-1.84916
C	-1.12423	-0.74795	-3.60200
H	0.02555	2.95781	-2.25208
H	-1.16154	3.00119	-3.56148
H	0.12211	1.79557	-3.57851
H	1.31341	-2.38410	-3.62417
H	2.25500	-3.80950	-3.16834
H	2.72857	-2.21589	-2.56531
H	-4.36699	-1.82112	-1.17265
H	-2.84475	-2.56633	-1.66559
H	-3.90385	-1.83346	-2.86976
H	-5.13304	-0.06027	-0.12076
H	-5.26173	1.46332	-0.98940
H	-4.60049	1.44781	0.63668
H	1.36688	-2.54227	2.24933
H	0.73939	-2.62359	3.90013
H	1.67042	-1.21643	3.37009

H	3.39141	3.23118	-1.11942
H	2.90462	4.79567	-0.46408
H	1.78192	3.88664	-1.49258
H	3.08865	2.95643	3.12682
H	3.48769	4.42776	2.22150
H	4.25819	2.88899	1.79416
H	2.06591	1.67764	-2.26481
H	1.55581	0.00311	-2.32757
H	2.71099	0.61246	-3.52600
H	-4.67713	-0.57424	1.97130
H	-4.24869	-2.09629	2.74228
H	-4.17795	-1.95768	0.99147
H	-0.09958	-0.44745	-3.83271
H	-1.71945	-0.59470	-4.51324
H	-1.11723	-1.81993	-3.39405
H	-3.46939	3.68520	-1.24992
H	-1.74730	3.84432	-0.90711
H	-2.85449	3.27606	0.34747
H	-2.47823	2.37898	2.52814
H	-2.93120	1.60161	4.04139
H	-3.92185	1.36714	2.60829
H	-2.25643	-3.58685	0.93899
H	-2.05718	-3.90474	2.66257
H	-0.64329	-3.84938	1.60672
H	-0.40266	4.01793	0.91586
H	0.78379	5.00596	1.80428
H	0.10697	3.55922	2.55235
H	4.85830	0.03118	-3.26166
H	-1.14283	-4.09484	-2.58283
H	-1.17671	-4.69190	-0.91456
H	-0.03417	-5.37428	-2.08014
H	4.19331	0.54995	2.59265
H	5.48039	-0.65640	2.47572
H	3.78687	-1.15101	2.39248
H	2.49917	-4.79011	-0.46874
H	1.43709	-4.36119	0.87499
H	2.70784	-3.23591	0.34685
H	1.09956	1.16288	3.41677
H	0.21418	0.58244	4.82748
H	-0.39647	1.96486	3.92357
H	6.59692	-0.83974	0.54879
H	7.82031	-1.40948	-1.51545
H	7.79841	0.19774	-2.24390
H	7.06861	-1.16077	-3.10000

100

U (VI) intermediate

C	-0.52299	-1.31530	2.64852
C	-0.83902	0.03837	2.96608
C	-2.18441	0.28280	2.57824
C	-2.70693	-0.92912	2.03020
C	-1.67102	-1.90577	2.05718
C	0.01770	0.94253	3.79572

C	-2.94831	1.53544	2.89751
C	-4.15252	-1.29100	1.85542
C	-1.86955	-3.36339	1.77536
C	0.67969	-2.05168	3.14896
U	-0.78190	-0.06723	0.12859
N	-0.12447	-1.67833	-0.66900
Si	0.63528	-3.16840	-1.29195
C	-0.69895	-4.43256	-1.77372
N	0.73781	1.55169	0.39572
Si	1.22834	3.24050	0.76446
C	2.79371	3.29338	1.83119
P	1.78975	0.27956	0.95199
C	3.27120	0.02587	-0.14373
C	3.39001	0.29526	-1.52748
C	4.61111	0.10486	-2.17790
C	5.74327	-0.36449	-1.51137
C	5.61466	-0.65295	-0.15387
C	4.41642	-0.46592	0.54111
C	2.21620	0.75432	-2.33841
C	7.05686	-0.53132	-2.22664
C	4.40858	-0.80647	2.01363
C	1.76248	-3.95184	0.01509
C	1.72856	-2.84464	-2.80525
C	-0.12028	4.18112	1.71201
C	1.62439	4.26659	-0.78580
C	-2.37747	1.91569	-1.28757
C	-1.46699	1.39650	-2.24634
C	-1.80073	0.03175	-2.47417
C	-2.92547	-0.29044	-1.66257
C	-3.29942	0.88736	-0.94941
C	-0.59378	2.23430	-3.12989
C	-2.55830	3.36536	-0.95559
C	-4.62073	1.16354	-0.29666
C	-3.68318	-1.58483	-1.74750
C	-1.28026	-0.82247	-3.58847
H	-0.01203	2.97272	-2.57671
H	-1.21827	2.78478	-3.84722
H	0.10308	1.63008	-3.71245
H	1.17692	-2.41273	-3.64343
H	2.15268	-3.79851	-3.14093
H	2.56004	-2.18044	-2.55343
H	-4.66164	-1.51155	-1.26643
H	-3.14732	-2.42431	-1.29051
H	-3.86274	-1.85640	-2.79454
H	-5.19136	0.25412	-0.10893
H	-5.22714	1.79117	-0.96405
H	-4.53019	1.70774	0.64669
H	0.99549	-2.84525	2.46947
H	0.45613	-2.51821	4.11893
H	1.52561	-1.37599	3.28932
H	2.36614	3.78676	-1.43048
H	2.05037	5.22049	-0.45207

H	0.74153	4.50110	-1.38684
H	2.64620	2.80365	2.79757
H	3.06236	4.34023	2.01836
H	3.63906	2.81000	1.33272
H	1.82412	1.70489	-1.97750
H	1.39596	0.03040	-2.27032
H	2.48661	0.86422	-3.39298
H	-4.80851	-0.42211	1.91226
H	-4.45344	-1.97379	2.66148
H	-4.35751	-1.80838	0.91483
H	-0.24170	-0.59233	-3.83634
H	-1.87359	-0.66255	-4.49990
H	-1.33445	-1.88488	-3.34445
H	-3.27180	3.83420	-1.64896
H	-1.62379	3.92560	-1.03472
H	-2.95793	3.51687	0.05209
H	-2.65134	2.39418	2.28384
H	-2.78955	1.82741	3.94203
H	-4.02351	1.39316	2.76643
H	-2.53520	-3.53178	0.92359
H	-2.32148	-3.86911	2.64060
H	-0.92429	-3.86623	1.56218
H	-1.10081	4.11310	1.23699
H	0.15252	5.24202	1.75769
H	-0.20962	3.82048	2.73992
H	4.67231	0.32132	-3.24353
H	-1.34236	-4.06562	-2.57893
H	-1.33450	-4.69038	-0.92187
H	-0.22198	-5.35467	-2.12580
H	4.08704	0.04305	2.62498
H	5.40973	-1.10569	2.33805
H	3.72733	-1.63300	2.24263
H	2.33183	-4.76941	-0.44232
H	1.20922	-4.36922	0.86106
H	2.47825	-3.21589	0.39216
H	1.04604	0.98401	3.41760
H	0.06517	0.57903	4.83107
H	-0.37790	1.95951	3.82390
H	6.47776	-1.03176	0.39120
H	7.68363	-1.28673	-1.74333
H	7.62524	0.40740	-2.23411
H	6.91062	-0.82825	-3.26995

100

U(VI) TS P - N bond formation

C	5.07294	7.45436	10.50443
C	4.74393	8.82988	10.66876
C	3.37238	9.00431	10.32996
C	2.84946	7.73129	9.96333
C	3.91380	6.78464	10.03012
C	5.62981	9.84212	11.32402
C	2.59299	10.27477	10.50711
C	1.40596	7.32659	9.94625

C	3.73864	5.30099	9.89015
C	6.31270	6.79987	11.02798
U	4.63651	8.42583	7.86510
N	6.06913	7.16979	7.24066
Si	6.91426	5.66364	6.79409
C	5.63745	4.25800	6.70814
N	6.29153	10.00226	7.89510
Si	6.77739	11.70988	8.04332
C	8.17605	11.93604	9.30641
P	7.33749	8.76234	8.42339
C	8.84091	8.69990	7.31789
C	8.91759	8.94904	5.92666
C	10.16395	8.98652	5.29607
C	11.35643	8.78097	5.99008
C	11.27211	8.53257	7.35878
C	10.04996	8.48841	8.03650
C	7.69552	9.16939	5.08431
C	12.68218	8.79684	5.27831
C	10.10390	8.22531	9.52712
C	8.24195	5.14994	8.04705
C	7.77478	5.71740	5.10451
C	5.29607	12.75099	8.60308
C	7.43184	12.48046	6.43517
C	2.77660	9.91172	6.26730
C	3.77530	9.43685	5.37143
C	3.64037	8.02256	5.27301
C	2.55845	7.62608	6.10954
C	2.00115	8.80214	6.69433
C	4.58631	10.31553	4.46609
C	2.40773	11.34917	6.49080
C	0.62939	8.94333	7.28286
C	1.97668	6.24250	6.16651
C	4.32333	7.14606	4.26799
H	5.08491	11.12754	5.00147
H	3.93810	10.77768	3.70904
H	5.35272	9.75329	3.93122
H	7.09114	5.98197	4.29392
H	8.17250	4.71797	4.89044
H	8.61256	6.41898	5.09728
H	2.75167	5.46974	6.16283
H	1.32926	6.04755	5.30037
H	1.36514	6.08881	7.06004
H	0.21091	7.99140	7.61093
H	-0.05070	9.34380	6.51783
H	0.59158	9.63987	8.12567
H	6.50245	5.83745	10.54777
H	6.21041	6.60973	12.10548
H	7.19466	7.42719	10.88030
H	8.34269	11.98222	6.09049
H	7.68587	13.52787	6.63865
H	6.70323	12.47427	5.61941
H	7.89860	11.59135	10.30589

H	8.42649	13.00125	9.37636
H	9.08151	11.40450	8.99650
H	7.33458	10.19462	5.17924
H	6.88124	8.51537	5.40076
H	7.91769	8.98576	4.02850
H	0.73158	8.17760	9.85015
H	1.16095	6.82973	10.89542
H	1.16748	6.61371	9.15190
H	4.39446	6.11109	4.60901
H	5.33579	7.48797	4.04265
H	3.76390	7.13646	3.32201
H	2.16122	11.56781	7.53549
H	1.52398	11.62136	5.89627
H	3.21191	12.02474	6.18835
H	3.13229	11.14923	10.12765
H	2.38333	10.46828	11.56770
H	1.62949	10.23694	9.99225
H	3.15404	5.02724	9.00521
H	3.20889	4.88580	10.75903
H	4.69981	4.78616	9.82375
H	4.41352	12.58038	7.98167
H	5.54827	13.81539	8.53596
H	5.02799	12.54083	9.64234
H	10.19784	9.17428	4.22388
H	4.92074	4.40853	5.89586
H	5.07833	4.14913	7.64037
H	6.15672	3.31189	6.51495
H	9.71355	9.06728	10.10756
H	11.13955	8.05568	9.83660
H	9.52284	7.34527	9.81758
H	8.62070	4.15933	7.76907
H	7.86143	5.08718	9.06925
H	9.08682	5.84284	8.03093
H	6.66191	9.75888	10.96908
H	5.64612	9.69056	12.41206
H	5.28817	10.86442	11.14541
H	12.18772	8.36953	7.92429
H	13.50846	8.97245	5.97303
H	12.71369	9.57381	4.50799
H	12.87132	7.83864	4.77864
16			
N3SiMe3			
N	-1.91480	4.30001	12.00322
N	-1.05269	4.33061	12.75173
N	-0.15724	4.26517	13.57871
Si	1.00326	5.59130	13.93422
C	0.04205	7.10405	14.52101
C	1.98175	5.98328	12.37023
C	2.09261	4.87665	15.28607
H	2.52258	5.10164	12.01372
H	2.71335	6.77436	12.56846
H	1.32530	6.32967	11.56568

H	2.59843	3.97090	14.93907
H	1.49979	4.61842	16.16839
H	2.85634	5.60110	15.58689
H	0.73082	7.91903	14.76950
H	-0.54749	6.87328	15.41319
H	-0.63982	7.46782	13.74575
2			
N2			
N	0.000000	0.000000	-0.007161
N	0.000000	0.000000	1.097161
80			
Complex 1			
C	0.01334	0.70495	-3.32868
C	0.24965	-0.61503	-2.85708
C	1.65650	-0.79929	-2.74764
C	2.29103	0.41127	-3.15648
C	1.27375	1.34919	-3.50138
C	-0.80290	-1.54826	-2.34064
C	2.32199	-1.99468	-2.13328
C	3.75994	0.71401	-3.09080
C	1.50170	2.81758	-3.70947
C	-1.34438	1.33995	-3.39840
U	1.27893	-0.61023	-5.45313
C	-0.37681	-2.30834	-5.41397
P	3.36792	-2.45191	-5.71341
C	2.87350	-4.22342	-5.50788
C	2.22426	-4.91012	-6.54740
C	1.83553	-6.24098	-6.40184
C	2.09423	-6.92267	-5.21389
C	2.74342	-6.26053	-4.17275
C	3.12389	-4.92694	-4.31652
Si	5.61135	-2.58488	-5.94810
C	6.33277	-0.82963	-6.01885
C	6.04679	-3.51419	-7.54683
C	6.41313	-3.50776	-4.49261
C	1.96911	0.30000	-7.94005
C	0.92265	-0.63822	-8.17398
C	-0.29187	-0.07574	-7.68877
C	0.00463	1.21279	-7.15818
C	1.40242	1.44546	-7.31318
C	-1.02670	2.22850	-6.76397
C	-1.66784	-0.63587	-7.89319
C	1.05138	-1.91259	-8.95465
C	3.37452	0.20401	-8.45368
C	2.13047	2.74142	-7.10202
H	-0.28073	-2.84708	-6.36955
H	-1.43951	-2.08136	-5.26696
H	-0.07334	-3.01418	-4.62498
H	4.36002	-0.19917	-3.10662
H	4.01086	1.25845	-2.17039
H	4.09216	1.34034	-3.92668
H	1.54897	3.44670	-6.50585

H	2.33844	3.22587	-8.06519
H	3.09803	2.60882	-6.60410
H	2.02530	-4.39091	-7.47981
H	3.62032	-4.42099	-3.49423
H	1.33270	-6.74714	-7.22222
H	2.04954	-2.34651	-8.85237
H	0.87471	-1.73056	-10.02356
H	0.32599	-2.66654	-8.63476
H	4.07198	0.78686	-7.84425
H	3.44139	0.59766	-9.47747
H	3.72911	-0.82922	-8.46817
H	5.56420	-4.49619	-7.56254
H	7.12965	-3.66497	-7.62770
H	5.71132	-2.95971	-8.42858
H	-1.32164	2.31352	-3.89167
H	-1.74453	1.49905	-2.38832
H	-2.07011	0.71289	-3.92826
H	6.07514	-0.25739	-5.12296
H	5.97154	-0.28264	-6.89371
H	7.42552	-0.88130	-6.08330
H	6.22189	-2.99798	-3.54345
H	7.49858	-3.56096	-4.63881
H	6.03318	-4.53028	-4.41392
H	-1.87784	1.77909	-6.24514
H	-1.42617	2.72971	-7.65601
H	-0.61804	3.00924	-6.11764
H	1.85463	-2.93254	-2.44740
H	2.26020	-1.94878	-1.03725
H	3.37617	-2.05227	-2.41136
H	2.95102	-6.78140	-3.24119
H	0.68128	3.29642	-4.25037
H	2.42538	3.02412	-4.25668
H	1.58508	3.32904	-2.74075
H	-1.72362	-1.49872	-2.92832
H	-1.06365	-1.29509	-1.30396
H	-0.46755	-2.58855	-2.34458
H	-1.66459	-1.72794	-7.93006
H	-2.09592	-0.27802	-8.83932
H	-2.35658	-0.33975	-7.09617
H	1.79332	-7.96063	-5.10025

96

1 - N3SiMe3 coordination

C	-0.58197	0.40369	-3.19946
C	-0.70716	-1.01658	-3.16720
C	0.48127	-1.54569	-2.58849
C	1.33450	-0.45911	-2.25301
C	0.67844	0.74597	-2.63169
C	-1.80430	-1.80450	-3.82085
C	0.87260	-2.98944	-2.49275
C	2.74195	-0.60368	-1.75784
C	1.28902	2.11599	-2.68151
C	-1.42868	1.33969	-4.00954

U	-0.83887	-0.25928	-0.49492
N	1.03010	1.41888	0.37273
N	1.58300	2.33485	0.78115
N	2.08809	3.28754	1.32411
P	0.69012	-1.61855	1.46501
Si	2.68342	-1.56879	2.52352
C	3.75441	-3.04590	1.98302
C	-1.96950	-2.37258	-0.54478
Si	3.43064	4.38120	0.78696
C	3.90732	5.25934	2.37418
C	2.72363	5.56284	-0.49612
C	4.83258	3.33836	0.08848
C	0.09484	-3.24860	2.11746
C	0.29015	-4.44007	1.39902
C	-0.14970	-5.66645	1.89539
C	-0.79600	-5.73431	3.12883
C	-0.99624	-4.56470	3.86009
C	-0.55716	-3.33949	3.35899
C	2.46725	-1.66826	4.41121
C	3.65490	0.02531	2.15122
C	-3.50357	0.20950	0.23092
C	-2.73582	0.32226	1.42379
C	-1.94364	1.50047	1.33158
C	-2.20375	2.10526	0.06552
C	-3.17713	1.31148	-0.60695
C	-4.64448	-0.73563	0.00588
C	-2.88493	-0.52991	2.64827
C	-1.15473	2.07816	2.47170
C	-1.70533	3.44334	-0.39879
C	-3.95639	1.70279	-1.82788
H	-1.28501	-3.17987	-0.83260
H	-2.83665	-2.41536	-1.21729
H	-2.32634	-2.61591	0.46597
H	-0.46587	1.34757	2.90924
H	-1.83092	2.40757	3.27150
H	-0.57411	2.95496	2.17353
H	0.60231	2.89858	-2.34134
H	1.58009	2.37256	-3.70910
H	2.19432	2.17570	-2.07133
H	0.79505	-4.40040	0.43850
H	-0.71733	-2.43466	3.93747
H	5.14901	2.57615	0.80583
H	5.69343	3.97974	-0.13110
H	4.54547	2.83888	-0.84176
H	0.01485	-6.57172	1.31593
H	1.37605	-3.20451	-1.54533
H	1.56616	-3.25435	-3.30241
H	0.01052	-3.65547	-2.57388
H	3.11230	0.31778	-1.30025
H	3.42380	-0.85366	-2.58233
H	2.81778	-1.39576	-1.00722
H	3.20738	-3.98438	2.11300

```
H       4.67176     -3.10037      2.58133
H       4.03790     -2.96380      0.92894
H      -3.45068      2.47135     -2.41531
H      -4.93343      2.11207     -1.53635
H      -4.15753      0.85474     -2.49042
H       3.15265      0.90679      2.56067
H       3.80222      0.16591      1.07681
H       4.64316     -0.04386      2.62104
H       1.92919     -0.79428      4.79154
H       3.44599     -1.70988      4.90418
H       1.90618     -2.56288      4.69570
H      -2.45224      0.97882     -4.13034
H      -1.00790      1.45122     -5.01899
H      -1.47732      2.34309     -3.57595
H       3.04722      5.78272      2.80177
H       4.69424      5.99615      2.18272
H       4.27847      4.54798      3.11745
H      -3.05746     -1.58179      2.40412
H      -3.73614     -0.19166      3.25573
H      -1.98966     -0.48523      3.27246
H      -1.49714     -4.60366      4.82449
H      -1.54626      3.47057     -1.48164
H      -0.76209      3.72138      0.07979
H      -2.42637      4.23776     -0.16322
H      -4.78892     -0.96420     -1.05436
H      -5.58238     -0.29559      0.37171
H      -4.50227     -1.68356      0.52939
H      -1.88594     -2.81048     -3.40295
H      -1.62252     -1.90884     -4.89903
H      -2.78198     -1.32490     -3.70592
H       2.38756      5.02811     -1.38928
H       3.49077      6.28224     -0.80324
H       1.87645      6.12315     -0.09010
H      -1.13933     -6.68973      3.51671
96
From 1 - N-P coupling TS
C      -0.73008      0.23819     -3.73484
C      -0.73954     -1.18708     -3.65817
C       0.47745     -1.60281     -3.05245
C       1.22526     -0.43606     -2.72567
C       0.48040      0.70063     -3.14869
C      -1.76715     -2.09177     -4.27289
C       0.97223     -3.01417     -2.94870
C       2.64121     -0.41274     -2.23700
C       0.97543      2.11596     -3.18740
C      -1.63205      1.07894     -4.58868
U      -0.95526     -0.35013     -1.01323
N       0.34408      1.46284     -0.15808
N       1.15847      1.97694      0.54165
N       1.94752      1.55924      1.43389
P       1.12008     -0.94825      1.00010
Si      3.16929     -1.91768      1.42149
```

C	3.18611	-3.66963	0.68458
C	-1.40364	-2.71305	-0.65688
Si	2.99330	2.79365	2.25400
C	3.87182	1.87910	3.64529
C	1.90382	4.17007	2.95182
C	4.23071	3.49883	1.01320
C	0.34340	-1.29067	2.64094
C	-0.16191	-2.57516	2.90745
C	-0.72027	-2.88203	4.14732
C	-0.80315	-1.90815	5.14219
C	-0.31888	-0.62593	4.88738
C	0.25272	-0.32122	3.65187
C	3.42136	-2.07534	3.29462
C	4.62770	-0.93595	0.70409
C	-3.69965	-0.53818	-0.48956
C	-3.10682	-0.12904	0.73870
C	-2.62010	1.19700	0.57092
C	-2.90962	1.60706	-0.76420
C	-3.59751	0.54210	-1.41202
C	-4.52583	-1.77203	-0.69727
C	-3.20341	-0.87326	2.03684
C	-2.11176	2.09093	1.66361
C	-2.67511	2.98894	-1.30135
C	-4.37393	0.62205	-2.69341
H	-0.50479	-3.33510	-0.74080
H	-2.13003	-3.08219	-1.39722
H	-1.83354	-2.90492	0.33541
H	-1.52786	1.54326	2.40847
H	-2.95095	2.56242	2.19352
H	-1.48202	2.89241	1.27040
H	0.17002	2.83820	-3.02499
H	1.42449	2.34388	-4.16416
H	1.73610	2.29942	-2.42522
H	-0.11936	-3.33647	2.13359
H	0.64233	0.67322	3.45854
H	4.86658	2.71222	0.59600
H	4.87930	4.24045	1.49252
H	3.71020	3.99029	0.18544
H	-1.09938	-3.88391	4.33223
H	1.53667	-3.18584	-2.02807
H	1.63581	-3.25337	-3.79065
H	0.15224	-3.73580	-2.96491
H	2.82678	0.40333	-1.53300
H	3.34259	-0.28847	-3.07378
H	2.90073	-1.34864	-1.73716
H	2.34135	-4.26104	1.04991
H	4.10995	-4.18391	0.97520
H	3.13998	-3.65349	-0.40809
H	-4.07029	1.46833	-3.31143
H	-5.44230	0.75205	-2.47318
H	-4.28964	-0.28511	-3.30055
H	4.55184	0.12222	0.96471

H	4.69257	-1.01420	-0.38309
H	5.56027	-1.32885	1.12568
H	3.37590	-1.10623	3.79670
H	4.40782	-2.51513	3.48407
H	2.66487	-2.72232	3.74529
H	-2.58386	0.58716	-4.79547
H	-1.15262	1.27042	-5.55871
H	-1.84468	2.05592	-4.14508
H	3.15433	1.42359	4.33405
H	4.48743	2.58201	4.21675
H	4.52853	1.09275	3.26403
H	-3.09208	-1.95284	1.90650
H	-4.18628	-0.70425	2.49796
H	-2.44797	-0.55169	2.75683
H	-0.38037	0.14144	5.65513
H	-2.78247	3.02908	-2.38839
H	-1.67440	3.35691	-1.05259
H	-3.39704	3.70245	-0.88195
H	-4.49669	-2.11947	-1.73472
H	-5.57944	-1.57698	-0.45314
H	-4.19227	-2.59867	-0.06576
H	-1.82039	-3.05552	-3.75957
H	-1.53348	-2.29689	-5.32637
H	-2.76895	-1.65258	-4.24890
H	1.36658	4.68712	2.15105
H	2.51079	4.90950	3.48595
H	1.16511	3.76854	3.65207
H	-1.24413	-2.14592	6.10630

96

From 1 - N-P coupling product

C	-0.63372	0.48398	-3.74570
C	-0.64679	-0.94160	-3.67463
C	0.56573	-1.36532	-3.06095
C	1.31780	-0.20164	-2.73019
C	0.57946	0.93793	-3.15192
C	-1.67061	-1.86208	-4.27477
C	1.04260	-2.78480	-2.98935
C	2.71721	-0.17564	-2.19258
C	1.06401	2.35700	-3.14799
C	-1.54672	1.34645	-4.56780
U	-0.90238	-0.11458	-1.05335
N	0.32767	1.48841	-0.16196
N	1.18111	2.06708	0.47412
N	1.92732	1.26772	1.47417
P	1.54239	-0.42172	1.49356
Si	3.48774	-1.66220	1.75652
C	3.14814	-3.30133	0.86233
C	-0.91361	-2.46289	-0.39011
Si	2.99991	2.44733	2.32776
C	3.86307	1.63506	3.79853
C	1.94309	3.87865	2.97013
C	4.26474	3.13125	1.10242

C	0.68901	-0.75538	3.09240
C	0.29643	-2.07325	3.38096
C	-0.36796	-2.37650	4.56702
C	-0.67330	-1.36675	5.48023
C	-0.31140	-0.05216	5.19240
C	0.36515	0.25095	4.01046
C	3.89184	-2.07559	3.56173
C	4.95176	-0.80404	0.91440
C	-3.57288	-0.43282	-0.47740
C	-2.99609	-0.01524	0.75364
C	-2.58029	1.33897	0.60891
C	-2.91468	1.76321	-0.71029
C	-3.54036	0.67507	-1.38008
C	-4.27129	-1.73577	-0.73629
C	-2.99834	-0.79162	2.03693
C	-2.08918	2.23274	1.70856
C	-2.74371	3.16390	-1.22082
C	-4.31541	0.73755	-2.66395
H	0.08635	-2.91336	-0.42415
H	-1.56464	-3.07005	-1.03851
H	-1.28171	-2.58258	0.63833
H	-1.55988	1.67019	2.48203
H	-2.93082	2.74328	2.19694
H	-1.40937	3.00215	1.33397
H	0.23995	3.06680	-3.03204
H	1.57296	2.59857	-4.09152
H	1.76705	2.54349	-2.33291
H	0.49475	-2.86717	2.66468
H	0.63723	1.27797	3.78894
H	4.90991	2.34578	0.69879
H	4.90196	3.88239	1.58240
H	3.74664	3.60909	0.26599
H	-0.65920	-3.40336	4.77192
H	1.76922	-2.93694	-2.18665
H	1.53415	-3.07474	-3.92812
H	0.22187	-3.48683	-2.81998
H	2.87467	0.66024	-1.50462
H	3.44945	-0.07314	-3.00509
H	2.95809	-1.09874	-1.65880
H	2.30629	-3.84186	1.30517
H	4.03137	-3.94730	0.92689
H	2.92108	-3.13960	-0.19460
H	-4.03775	1.59951	-3.27312
H	-5.38922	0.82728	-2.45035
H	-4.19341	-0.16011	-3.27844
H	5.28810	0.07433	1.46995
H	4.70332	-0.48990	-0.10197
H	5.79076	-1.50691	0.85637
H	4.20702	-1.20118	4.13438
H	4.70569	-2.80993	3.58328
H	3.02585	-2.51460	4.06487
H	-2.48691	0.84503	-4.80488

```
H      -1.06751      1.59876     -5.52354
H      -1.78765      2.29427     -4.07653
H       3.15918      1.09164      4.43515
H       4.31708      2.42763      4.40471
H       4.66372      0.95383      3.50146
H      -3.04737     -1.86932      1.86262
H      -3.87373     -0.52297      2.64361
H      -2.11330     -0.59597      2.64754
H      -0.55452      0.74475      5.89053
H      -2.88563      3.22615     -2.30293
H      -1.74826      3.55679     -0.99084
H      -3.47599      3.84144     -0.76186
H      -4.18737     -2.04568     -1.78321
H      -5.34410     -1.66023     -0.51152
H      -3.86501     -2.54413     -0.12315
H      -1.90222     -2.70535     -3.61488
H      -1.31351     -2.28872     -5.22167
H      -2.60884     -1.34465     -4.49261
H       1.41168      4.36132      2.14615
H       2.58245      4.62621      3.45365
H       1.20250      3.54836      3.70494
H      -1.19714     -1.60200      6.40230
96
From 1 - N-N activation TS
C      -1.42061      3.41721      8.51110
C      -0.70765      4.61711      8.79560
C      -1.49564      5.71564      8.35532
C      -2.70042      5.20008      7.79535
C      -2.65832      3.77780      7.90615
C       0.69986      4.71341      9.30347
C      -1.06127      7.14980      8.29971
C      -3.65408      6.00618      6.96246
C      -3.65313      2.80298      7.34411
C      -0.89068      2.01851      8.61177
C       2.97920      4.21693     12.36599
Si      1.54202      3.35595     13.24987
C       1.23528      1.67500     12.42243
P      -0.42039      4.57007     12.99159
C      -1.25014      4.22277     14.60344
C      -1.60752      2.89999     14.91247
C      -2.24265      2.59441     16.11408
C      -2.55309      3.60690     17.02260
C      -2.22543      4.92615     16.71502
C      -1.57768      5.23125     15.51753
U      -2.90228      4.66373     10.50338
N      -1.73616      6.39933     11.30120
N      -0.91024      7.03849     11.88554
C      -2.82403      2.34040     11.22823
C       1.98217      3.01798     15.06276
N      -0.06635      6.24043     12.94497
Si      0.97301      7.46856     13.74142
C       2.18449      8.17535     12.47341
```

C	1.91107	6.73789	15.21144
C	-0.11726	8.88100	14.37439
C	-4.97921	4.73665	12.32931
C	-4.61714	6.10087	12.13934
C	-4.98250	6.47228	10.81248
C	-5.57156	5.34074	10.18223
C	-5.55187	4.26029	11.11788
C	-4.15063	7.04429	13.20787
C	-4.87298	7.86268	10.25893
C	-6.36669	5.33566	8.90892
C	-6.20294	2.92500	10.90407
C	-4.94104	4.00234	13.63613
H	-1.80999	1.92142	11.21930
H	-3.44068	1.70886	10.56889
H	-3.21403	2.21466	12.24734
H	-3.56318	6.53213	13.97427
H	-5.00847	7.50956	13.71283
H	-3.53336	7.84819	12.79944
H	-1.90542	7.83519	8.41749
H	-0.58929	7.37570	7.33344
H	-0.34023	7.38729	9.08500
H	-1.40342	2.10328	14.20102
H	-1.32917	6.26094	15.28013
H	2.84939	7.40508	12.07213
H	2.80405	8.95981	12.92226
H	1.63182	8.61542	11.63810
H	-2.50630	1.56350	16.33566
H	-0.13791	1.91702	9.39784
H	-0.41409	1.71833	7.66865
H	-1.68006	1.29261	8.82426
H	0.83244	5.55657	9.98773
H	1.40695	4.85021	8.47403
H	0.99708	3.80414	9.83184
H	0.42635	1.12192	12.90894
H	2.14262	1.06317	12.48614
H	0.97543	1.78806	11.36681
H	-6.13080	6.18849	8.27028
H	-7.43968	5.39231	9.13744
H	-6.22111	4.42483	8.31952
H	2.73625	4.44696	11.32626
H	3.85029	3.55169	12.37196
H	3.26365	5.14746	12.86200
H	2.27784	3.92173	15.59920
H	2.81929	2.31071	15.09714
H	1.13759	2.57033	15.59379
H	-4.56609	5.45427	6.72797
H	-3.18419	6.27202	6.00592
H	-3.94513	6.94579	7.44207
H	1.24546	6.19422	15.88789
H	2.35968	7.56336	15.77646
H	2.72245	6.07077	14.91009
H	-4.95781	2.91857	13.49702

H	-5.81776	4.26287	14.24436
H	-4.05590	4.24507	14.22911
H	-2.47198	5.72453	17.41036
H	-5.04704	7.88892	9.18012
H	-3.88550	8.29466	10.44947
H	-5.61492	8.52801	10.71988
H	-6.09701	2.57627	9.87131
H	-7.28004	2.97375	11.11482
H	-5.77722	2.15604	11.55342
H	-3.83800	1.96476	8.02458
H	-3.29415	2.37190	6.39999
H	-4.61591	3.27566	7.13234
H	-0.69651	9.31210	13.55380
H	0.50615	9.67125	14.80835
H	-0.81780	8.54735	15.14615
H	-3.05352	3.36979	17.95718

96
From 1 - N-N activation product

C	0.94322	-1.09437	-2.76249
C	1.35980	0.25131	-2.54485
C	0.35075	1.10802	-3.07302
C	-0.67923	0.29840	-3.62946
C	-0.32148	-1.06776	-3.42175
C	2.70242	0.70024	-2.03849
C	0.46337	2.59476	-3.24777
C	-1.72169	0.79506	-4.58680
C	-1.04877	-2.25699	-3.98125
C	1.78506	-2.31483	-2.53980
C	4.62790	-1.29339	0.52551
Si	3.16768	-1.85056	1.59835
C	2.66927	-3.61555	1.08377
P	1.52874	-0.18075	1.44317
C	0.60410	-0.60380	3.00733
C	0.11532	-1.89381	3.26143
C	-0.53202	-2.18960	4.45959
C	-0.72117	-1.19384	5.41930
C	-0.25052	0.09548	5.17223
C	0.41255	0.38540	3.97918
U	-0.87133	-0.19390	-0.91484
N	0.16606	1.90134	-0.17367
N	0.82929	2.71120	0.22163
C	-0.82105	-2.51666	-0.29474
C	3.71636	-1.93959	3.41124
N	2.32596	1.19172	1.62396
Si	3.35644	2.39086	2.27888
C	4.37956	3.18383	0.88466
C	4.59314	1.76473	3.58775
C	2.38397	3.80306	3.11285
C	-2.99089	-0.11126	0.86237
C	-2.70129	1.25622	0.59536
C	-3.04572	1.52025	-0.76527
C	-3.55632	0.31745	-1.33149

C	-3.50394	-0.69700	-0.32866
C	-2.32998	2.27731	1.63126
C	-3.04620	2.87639	-1.40827
C	-4.32518	0.18310	-2.61294
C	-4.11097	-2.06310	-0.45360
C	-2.96882	-0.75099	2.21636
H	0.21473	-2.87426	-0.23528
H	-1.34696	-3.14972	-1.02503
H	-1.28233	-2.70052	0.68388
H	-1.60065	1.89412	2.35053
H	-3.21892	2.57672	2.20192
H	-1.91442	3.18397	1.18552
H	-0.49781	3.10326	-3.12630
H	0.82499	2.83351	-4.25703
H	1.16478	3.03928	-2.53863
H	0.24683	-2.67785	2.52044
H	0.80849	1.37984	3.79205
H	5.00455	2.44132	0.37699
H	5.03959	3.96625	1.27675
H	3.72569	3.64008	0.13443
H	-0.89049	-3.19931	4.64471
H	2.44957	-2.20011	-1.68123
H	2.41781	-2.50768	-3.41648
H	1.18028	-3.20998	-2.37405
H	2.71305	0.99136	-0.98090
H	3.05763	1.55995	-2.61624
H	3.44244	-0.09431	-2.16371
H	1.88766	-4.02850	1.72748
H	3.55192	-4.25861	1.18842
H	2.33049	-3.68560	0.04756
H	-4.15211	1.01888	-3.29181
H	-5.40168	0.16152	-2.39670
H	-4.09759	-0.74160	-3.15306
H	4.44511	-1.46149	-0.53921
H	5.52661	-1.85461	0.80472
H	4.82369	-0.22864	0.67441
H	4.00181	-0.95572	3.78801
H	4.58243	-2.60643	3.49766
H	2.91678	-2.32974	4.04663
H	-2.52382	0.07178	-4.74445
H	-1.26138	0.98121	-5.56680
H	-2.17440	1.73860	-4.26883
H	4.07822	1.33225	4.45223
H	5.20624	2.59830	3.95111
H	5.27157	1.00788	3.18079
H	-2.77193	-1.82433	2.16683
H	-3.94409	-0.62392	2.70563
H	-2.21567	-0.30857	2.87082
H	-0.38806	0.87610	5.91661
H	-3.02953	2.81220	-2.49933
H	-2.18930	3.48229	-1.09877
H	-3.94954	3.43712	-1.13410

H	-3.98124	-2.48391	-1.45572
H	-5.19153	-2.02519	-0.25985
H	-3.67644	-2.76920	0.25772
H	-0.82579	-3.16963	-3.42263
H	-0.75976	-2.43707	-5.02519
H	-2.13555	-2.12265	-3.97200
H	1.64716	4.23426	2.42872
H	3.06743	4.60402	3.41910
H	1.85564	3.46517	4.01102
H	-1.22657	-1.42338	6.35344

94

From 1 - SiMe3 transf. adduct

C	1.54054	0.90708	-2.54493
C	1.21932	-0.46531	-2.74145
C	-0.07174	-0.53865	-3.33410
C	-0.54495	0.79095	-3.50933
C	0.44210	1.68626	-3.00804
C	2.18362	-1.59921	-2.56960
C	-0.71370	-1.76746	-3.90708
C	-1.68664	1.18181	-4.39977
C	0.43641	3.17142	-3.23080
C	2.90786	1.40022	-2.17712
U	-0.42453	0.31934	-0.65052
C	-0.72606	-2.04633	-0.60904
N	1.16830	0.37005	1.00081
P	1.95038	-0.69010	2.18001
Si	3.33134	-2.36322	1.42854
C	3.98204	-3.02185	3.09108
Si	1.78233	1.98814	1.50573
C	1.30517	2.46183	3.28536
C	1.07208	3.31459	0.32376
C	3.66807	2.18811	1.43155
C	0.69348	-1.63999	3.15469
C	0.09098	-2.84235	2.75340
C	-0.77083	-3.53484	3.60469
C	-1.04450	-3.03943	4.87909
C	-0.44439	-1.85396	5.30134
C	0.41902	-1.16779	4.44895
C	4.78658	-1.54299	0.52246
C	2.73748	-3.87442	0.43269
C	-2.45690	0.61371	1.23128
C	-2.29967	1.91431	0.66880
C	-2.78541	1.87917	-0.67035
C	-3.24391	0.55597	-0.93082
C	-3.04725	-0.22132	0.24543
C	-3.02325	3.09988	-1.51107
C	-2.03148	3.16534	1.45515
C	-2.27301	0.26662	2.67700
C	-3.63288	-1.58085	0.47926
C	-4.03771	0.08490	-2.11360
H	0.24324	-2.52821	-0.80147
H	-1.44748	-2.46599	-1.31973

H	-1.05110	-2.34540	0.39401
H	-1.22411	3.04794	2.18135
H	-2.92963	3.45221	2.01949
H	-1.77655	4.01109	0.81138
H	-0.56647	3.59904	-3.14337
H	0.79571	3.40993	-4.24141
H	1.08281	3.70393	-2.52935
H	0.29696	-3.24208	1.76703
H	0.89706	-0.25319	4.78916
H	0.87616	4.22535	0.89967
H	0.12627	3.07146	-0.17561
H	1.80318	3.56388	-0.44820
H	-1.22381	-4.46551	3.27249
H	2.69412	-1.57059	-1.60326
H	2.96308	-1.56590	-3.34307
H	1.68836	-2.56965	-2.65406
H	2.91531	2.46533	-1.93567
H	3.60225	1.25972	-3.01711
H	3.32312	0.86056	-1.32189
H	2.09397	-4.51612	1.04120
H	3.61600	-4.46513	0.14562
H	2.19730	-3.61268	-0.47884
H	-4.04888	0.81979	-2.91986
H	-5.08227	-0.09174	-1.82392
H	-3.66476	-0.85945	-2.52595
H	4.51599	-1.14077	-0.45721
H	5.58034	-2.28327	0.36963
H	5.19878	-0.72547	1.12089
H	4.42149	-2.22229	3.69432
H	4.75620	-3.77603	2.90795
H	3.18506	-3.49070	3.67553
H	-2.47548	0.42740	-4.42066
H	-1.32865	1.29454	-5.43297
H	-2.13674	2.13706	-4.11941
H	3.92149	3.22005	1.70451
H	4.08156	1.99468	0.43887
H	4.15493	1.52234	2.14879
H	-2.09201	-0.79992	2.82730
H	-3.17050	0.53111	3.25361
H	-1.43163	0.80094	3.12271
H	-0.64266	-1.46675	6.29738
H	-3.28867	2.84575	-2.53896
H	-2.15392	3.76497	-1.54870
H	-3.85542	3.69037	-1.10458
H	-3.56607	-2.22215	-0.40379
H	-4.69714	-1.49324	0.73789
H	-3.14325	-2.10409	1.30462
H	-0.38460	-2.67730	-3.39969
H	-0.46245	-1.87707	-4.97077
H	-1.80576	-1.73520	-3.83985
H	0.27239	2.21215	3.54174
H	1.43699	3.54156	3.42443

H	1.96038	1.94990	3.99536
H	-1.71413	-3.57984	5.54291

94

From 1 - SiMe3 transf. TS

C	1.86187	0.50048	-2.39345
C	1.46086	-0.83585	-2.67305
C	0.20211	-0.79069	-3.33440
C	-0.17243	0.57596	-3.46637
C	0.84303	1.37405	-2.86742
C	2.32544	-2.04586	-2.48468
C	-0.49866	-1.94829	-3.98282
C	-1.25692	1.08314	-4.37027
C	0.93318	2.87139	-2.95096
C	3.22371	0.89480	-1.89997
U	-0.26583	-0.03178	-0.64262
C	-0.65372	-2.42730	-0.62364
N	1.21842	-0.12140	0.93137
P	2.20140	-0.76031	2.12603
Si	3.86385	-2.31502	1.66003
C	4.71240	-2.60249	3.33230
Si	2.29984	1.54355	2.11876
C	1.43022	1.97020	3.75402
C	1.79025	2.81992	0.80615
C	4.17086	1.89392	2.30359
C	1.30220	-1.71475	3.42255
C	0.32684	-2.65965	3.07818
C	-0.30089	-3.41696	4.06519
C	0.03431	-3.23733	5.40769
C	1.00056	-2.29643	5.76009
C	1.63017	-1.53807	4.77363
C	5.15230	-1.62155	0.45715
C	3.13028	-3.94042	1.02337
C	-2.32615	0.32043	1.19427
C	-2.05159	1.63199	0.70667
C	-2.50746	1.70733	-0.64128
C	-3.05693	0.44075	-0.98500
C	-2.94930	-0.41369	0.14963
C	-2.60627	2.98108	-1.42914
C	-1.68012	2.81023	1.56043
C	-2.18304	-0.12390	2.61919
C	-3.62648	-1.74476	0.27848
C	-3.82269	0.08480	-2.22570
H	0.29466	-2.96533	-0.76868
H	-1.33749	-2.77488	-1.41028
H	-1.08240	-2.77675	0.32517
H	-0.94169	2.55944	2.32563
H	-2.56638	3.19422	2.08545
H	-1.27992	3.64070	0.97133
H	-0.05138	3.32921	-3.07889
H	1.54149	3.18191	-3.81186
H	1.38705	3.31814	-2.06128
H	0.05801	-2.79555	2.03507

H	2.38036	-0.80402	5.05607
H	1.98441	3.82641	1.19997
H	0.73345	2.75736	0.53963
H	2.39125	2.69084	-0.09534
H	-1.05269	-4.15030	3.78528
H	2.78408	-2.07302	-1.49218
H	3.14444	-2.06263	-3.21766
H	1.76163	-2.97363	-2.61072
H	3.36628	1.97827	-1.94072
H	4.00367	0.45057	-2.53244
H	3.41453	0.56921	-0.87238
H	2.42585	-4.35724	1.74843
H	3.93358	-4.67022	0.86942
H	2.60577	-3.80751	0.07402
H	-3.75890	0.86556	-2.98591
H	-4.88717	-0.05357	-1.99356
H	-3.47838	-0.85179	-2.68020
H	4.74330	-1.40673	-0.53216
H	5.95230	-2.36127	0.33561
H	5.60386	-0.70587	0.84991
H	5.12808	-1.67382	3.73519
H	5.54016	-3.30840	3.19808
H	4.02336	-3.02110	4.07022
H	-2.08185	0.37348	-4.46619
H	-0.85997	1.24504	-5.38249
H	-1.67295	2.03827	-4.03977
H	4.33786	2.97077	2.42929
H	4.72624	1.58445	1.41176
H	4.59339	1.37126	3.16715
H	-2.14560	-1.21217	2.70737
H	-3.03220	0.22200	3.22509
H	-1.27267	0.26348	3.08374
H	1.26100	-2.14687	6.80455
H	-2.84950	2.79682	-2.47775
H	-1.68096	3.56635	-1.40313
H	-3.39998	3.62530	-1.02673
H	-3.55832	-2.33593	-0.63899
H	-4.69413	-1.61137	0.50179
H	-3.20258	-2.34721	1.08596
H	-0.25578	-2.89653	-3.49709
H	-0.21315	-2.03914	-5.03981
H	-1.58794	-1.84075	-3.95631
H	0.46704	1.46736	3.86771
H	1.25798	3.05097	3.80963
H	2.06076	1.67949	4.59961
H	-0.45820	-3.82682	6.17612

94

Complex 3

C	1.24739	0.80170	-2.75462
C	1.05222	-0.58645	-3.00436
C	-0.18432	-0.74087	-3.68933
C	-0.74808	0.55258	-3.87459

C	0.13477	1.50615	-3.29398
C	2.06195	-1.66861	-2.77176
C	-0.70186	-2.00597	-4.30669
C	-1.92920	0.83683	-4.75528
C	0.04193	3.00174	-3.39853
C	2.48432	1.43070	-2.18383
U	-0.77435	0.05492	-1.03460
C	-1.32278	-2.32183	-1.06075
N	0.67820	-0.11499	0.49837
P	1.63749	-0.35539	1.76812
Si	3.47031	-1.74960	1.37472
C	4.30985	-2.23126	3.00674
Si	2.33774	1.63283	2.77741
C	1.05536	2.26648	4.02007
C	2.53652	2.86444	1.35962
C	4.00364	1.44203	3.67137
C	0.83725	-1.37843	3.08643
C	-0.16948	-2.27034	2.70059
C	-0.75355	-3.12145	3.63783
C	-0.34255	-3.08709	4.96988
C	0.66064	-2.20131	5.36219
C	1.25043	-1.35473	4.42445
C	4.75992	-0.90651	0.26861
C	2.73610	-3.29730	0.57558
C	-2.56938	0.77978	0.96999
C	-2.31315	1.96993	0.22936
C	-2.95857	1.85185	-1.03615
C	-3.56902	0.56820	-1.09202
C	-3.33661	-0.08982	0.14957
C	-3.19883	2.99486	-1.97768
C	-1.66538	3.21540	0.76218
C	-2.25943	0.57627	2.42200
C	-3.98496	-1.37273	0.57519
C	-4.45536	0.03714	-2.18113
H	-0.43361	-2.91129	-1.33144
H	-2.10060	-2.57091	-1.79857
H	-1.67887	-2.71044	-0.09712
H	-0.92576	2.98792	1.53494
H	-2.40376	3.89274	1.21434
H	-1.15527	3.78587	-0.02189
H	-0.91436	3.31839	-3.81977
H	0.82879	3.39788	-4.05402
H	0.15524	3.50806	-2.43186
H	-0.49309	-2.27913	1.66330
H	2.04031	-0.67941	4.74329
H	2.77446	3.85616	1.76018
H	1.61205	2.93665	0.78173
H	3.33847	2.56921	0.67774
H	-1.53273	-3.81179	3.32582
H	2.68236	-1.46005	-1.89733
H	2.73761	-1.77158	-3.63271
H	1.59006	-2.64375	-2.61819

H	2.31543	2.47975	-1.92364
H	3.31451	1.40723	-2.90369
H	2.81497	0.92138	-1.27465
H	2.21011	-3.89494	1.32558
H	3.52833	-3.91459	0.13809
H	2.02475	-3.03802	-0.21135
H	-4.42803	0.66984	-3.07133
H	-5.50166	-0.00992	-1.85064
H	-4.17667	-0.97818	-2.48755
H	4.36434	-0.62939	-0.71079
H	5.59440	-1.59882	0.10715
H	5.16592	-0.00557	0.73926
H	4.76892	-1.37852	3.51292
H	5.10389	-2.95427	2.78508
H	3.60487	-2.70611	3.69396
H	-2.75353	0.13638	-4.58996
H	-1.64972	0.74921	-5.81439
H	-2.31987	1.84719	-4.61395
H	4.31647	2.43012	4.02917
H	4.78777	1.06999	3.00609
H	3.94817	0.78089	4.54108
H	-2.29427	-0.47800	2.70496
H	-2.98434	1.10752	3.05463
H	-1.26476	0.94316	2.68610
H	0.98414	-2.16748	6.39914
H	-3.40975	2.66358	-2.99703
H	-2.35270	3.68561	-2.02098
H	-4.06861	3.58101	-1.64867
H	-4.07614	-2.07928	-0.25420
H	-4.99699	-1.18907	0.96214
H	-3.42358	-1.87402	1.36908
H	-0.39070	-2.89299	-3.74874
H	-0.33354	-2.12067	-5.33579
H	-1.79528	-2.02167	-4.35542
H	0.10868	2.51689	3.53570
H	1.44260	3.17902	4.48834
H	0.84746	1.54191	4.81162
H	-0.80189	-3.74732	5.70037

89

Complex 2

C	1.00507	-1.56329	-3.68552
C	1.88526	-0.71114	-2.96390
C	1.26164	0.56254	-2.83591
C	-0.01786	0.49230	-3.46307
C	-0.16933	-0.82306	-3.99501
C	3.13380	-1.16111	-2.27124
C	1.75545	1.71506	-2.01075
C	-1.09639	1.52316	-3.30263
C	-1.41226	-1.42921	-4.57888
C	1.16909	-3.04140	-3.87103
U	1.80788	0.38806	-5.51442
P	4.47615	1.23147	-5.07613

C	5.22624	1.75038	-3.45844
C	6.01881	0.86508	-2.68849
C	6.55662	1.29972	-1.46976
C	6.35280	2.58702	-0.98160
C	5.58736	3.45908	-1.75935
C	5.02656	3.07043	-2.97743
C	6.35094	-0.53709	-3.13281
C	6.92072	3.02508	0.34225
C	4.23551	4.08941	-3.75535
C	2.42521	-1.68962	-6.51070
Si	6.31105	1.28747	-6.44158
C	7.82704	1.86755	-5.45347
C	6.19834	2.53829	-7.87754
C	6.64532	-0.42660	-7.19389
C	1.78545	1.23244	-8.10833
C	1.64829	2.43294	-7.35232
C	0.35230	2.43406	-6.76403
C	-0.31674	1.24495	-7.16545
C	0.57525	0.49200	-7.98566
C	2.95113	0.87637	-8.98438
C	2.58146	3.60714	-7.36004
C	-0.24792	3.60132	-6.03558
C	-1.78595	0.97061	-7.03637
C	0.19869	-0.74398	-8.74961
H	2.99928	-1.47748	-7.42497
H	1.63923	-2.40917	-6.77239
H	3.11313	-2.18690	-5.81112
H	2.84556	1.74097	-1.94808
H	1.36811	1.65345	-0.98447
H	1.42611	2.67929	-2.41230
H	-1.20498	3.34734	-5.57580
H	-0.42922	4.43574	-6.72578
H	0.40654	3.98755	-5.24525
H	7.15992	0.60257	-0.89003
H	3.88697	0.76189	-8.42752
H	3.12230	1.65390	-9.73890
H	2.77542	-0.06115	-9.51709
H	3.61690	3.30748	-7.51953
H	2.54807	4.16087	-6.41826
H	2.30303	4.30815	-8.16027
H	5.79269	-0.77749	-7.78373
H	6.83073	-1.16536	-6.40907
H	7.52134	-0.39528	-7.85229
H	-2.18088	-0.67989	-4.77455
H	-1.84228	-2.16128	-3.88306
H	-1.21959	-1.96379	-5.51657
H	6.04187	3.55353	-7.49974
H	5.41301	2.31764	-8.60368
H	7.15488	2.52788	-8.41473
H	8.68244	1.93233	-6.13641
H	8.09504	1.20053	-4.63286
H	7.65346	2.86137	-5.03082

```
H      -2.01150     -0.09844     -7.02823
H      -2.32323      1.40190     -7.89205
H      -2.22189      1.41057     -6.13605
H       2.88362     -1.64816     -1.31858
H       3.80140     -0.32722     -2.04906
H       3.69391     -1.88617     -2.86730
H       5.42518      4.47753     -1.40984
H      -1.87853      1.42966     -4.05912
H      -0.70892      2.54468     -3.34957
H      -1.58343      1.41438     -2.32398
H       0.65493     -3.40369     -4.76534
H       0.74991     -3.58394     -3.01259
H       2.21898     -3.33348     -3.95571
H       1.07010     -1.34398     -9.02027
H      -0.32495     -0.48362     -9.67923
H      -0.47181     -1.39178     -8.17596
H       4.23268      5.05516     -3.24060
H       4.64697      4.23015     -4.76010
H       3.19648      3.77185     -3.89355
H       7.32800     -0.57214     -3.63016
H       6.40570     -1.21637     -2.27608
H       5.61339     -0.91430     -3.84427
H       7.35341      4.02919      0.27978
H       6.14425      3.05725      1.11660
H       7.70162      2.34217      0.68888
105
From 2 N3SiMe3 coordination
C       1.03292     -0.63825     -3.00028
C       1.58505      0.55367     -2.45616
C       0.70215      1.62766     -2.76165
C      -0.38671      1.10063     -3.51440
C      -0.18350     -0.30082     -3.65970
C       2.95308      0.68743     -1.85379
U      -0.69819      0.01119     -0.93959
C      -1.85103     -1.96122     -1.66944
C       0.99606      3.08448     -2.55088
C      -1.36912      1.90023     -4.31892
C      -0.94785     -1.19192     -4.59229
C       1.71727     -1.96975     -3.07716
N       1.07339      1.50548      0.46258
N       1.97164      1.87825      1.06602
N       2.95223      2.12089      1.73609
P       0.85599     -1.80609      0.65461
Si      2.71339     -3.12647      0.67454
C       4.29193     -2.21152      0.12373
Si      3.65085      3.73244      2.15769
C       4.21098      4.57937      0.57025
C       5.09092      3.28775      3.27419
C       2.33598      4.74748      3.04627
C       0.02993     -2.46874      2.19059
C      -0.78684     -3.62263      2.16260
C      -1.38896     -4.07888      3.34456
```

C	-1.20033	-3.44665	4.56864
C	-0.37146	-2.32136	4.59175
C	0.23833	-1.82484	3.43962
C	-1.02171	-4.43451	0.91463
C	-1.86315	-3.94564	5.82523
C	1.10570	-0.60045	3.56869
C	2.53887	-4.67445	-0.42308
C	3.03568	-3.73749	2.44568
C	-2.60771	0.46895	1.08828
C	-2.00900	1.73764	0.85054
C	-2.40193	2.17817	-0.44837
C	-3.25163	1.17905	-1.00611
C	-3.38192	0.13009	-0.05506
C	-2.20649	3.57539	-0.96025
C	-1.35357	2.59753	1.89131
C	-2.65133	-0.25318	2.40166
C	-4.38892	-0.97680	-0.12427
C	-4.06606	1.26971	-2.26326
H	-1.13246	-2.66317	-2.11285
H	-2.64895	-1.78987	-2.40597
H	-2.29945	-2.47387	-0.81011
H	-0.77271	2.01325	2.61013
H	-2.11285	3.14800	2.46404
H	-0.68569	3.34147	1.44835
H	0.10510	3.70095	-2.68779
H	1.74663	3.43842	-3.27045
H	1.38739	3.29346	-1.55015
H	4.94161	3.96703	0.03387
H	4.68075	5.54107	0.80460
H	3.36858	4.77564	-0.10027
H	-2.01748	-4.96749	3.29744
H	2.48983	-2.06752	-2.31200
H	2.20408	-2.10000	-4.05375
H	1.01967	-2.80199	-2.94786
H	3.10899	0.01966	-1.00350
H	3.13873	1.70918	-1.51253
H	3.72596	0.45477	-2.59802
H	2.31535	-4.41601	-1.46140
H	1.73623	-5.32129	-0.05838
H	3.47184	-5.25101	-0.40727
H	-3.81816	2.15549	-2.85069
H	-5.13538	1.32998	-2.02220
H	-3.93832	0.39297	-2.90901
H	4.43675	-1.29159	0.69847
H	4.29568	-1.95696	-0.93956
H	5.15413	-2.86412	0.30698
H	3.89460	-4.41929	2.43508
H	2.17459	-4.27457	2.85152
H	3.26765	-2.91234	3.12522
H	-2.29902	1.35583	-4.49739
H	-0.94357	2.13426	-5.30473
H	-1.62500	2.85433	-3.85164

H	4.74297	2.75222	4.16218
H	5.61758	4.18959	3.60259
H	5.80513	2.64734	2.74856
H	-3.56526	0.01371	2.95055
H	-1.80473	0.00172	3.04147
H	-2.64682	-1.34005	2.28612
H	-0.19345	-1.81373	5.53883
H	-2.34380	3.64689	-2.04154
H	-1.21425	3.97158	-0.72559
H	-2.93685	4.25656	-0.50188
H	-4.52933	-1.35140	-1.14125
H	-5.36522	-0.61815	0.23068
H	-4.11020	-1.82544	0.50579
H	-0.85482	-2.24574	-4.32078
H	-0.57116	-1.08342	-5.61862
H	-2.01582	-0.95538	-4.61408
H	1.48374	4.95989	2.39350
H	2.75735	5.70646	3.36753
H	1.96645	4.22492	3.93325
H	1.22613	-0.31695	4.61918
H	2.09544	-0.75607	3.13030
H	0.67107	0.25026	3.03269
H	-0.52074	-5.40781	0.98981
H	-2.08855	-4.63836	0.77067
H	-0.63917	-3.92328	0.03004
H	-1.14261	-4.04730	6.64439
H	-2.64163	-3.25289	6.16761
H	-2.33422	-4.92035	5.66926

105

From 2 - N-P coupling TS

C	0.45786	-0.94700	-3.38571
C	0.91489	0.33562	-2.97580
C	-0.06445	1.29465	-3.35158
C	-1.12352	0.60556	-4.01322
C	-0.79954	-0.78401	-4.02849
C	2.27723	0.65143	-2.43764
U	-1.28642	-0.19937	-1.34637
C	-2.03124	-2.49091	-1.63355
C	0.10257	2.78271	-3.24884
C	-2.17165	1.24834	-4.87376
C	-1.53506	-1.84195	-4.79601
C	1.25944	-2.21251	-3.33266
N	0.15885	1.15936	-0.11724
N	1.18160	1.34413	0.48226
N	1.95322	0.57523	1.13267
P	0.75783	-1.57900	0.61946
Si	2.73937	-2.77135	0.64596
C	4.15558	-1.79862	-0.16763
Si	3.27981	1.51739	1.97421
C	4.37578	2.33722	0.67130
C	4.30536	0.34294	3.03174
C	2.49393	2.83987	3.07048

C	-0.08005	-2.34429	2.11613
C	-0.64376	-3.63852	1.99539
C	-1.30643	-4.21904	3.08535
C	-1.44028	-3.57504	4.30867
C	-0.87955	-2.30181	4.41937
C	-0.21254	-1.67888	3.36329
C	-0.56681	-4.47941	0.74716
C	-2.17004	-4.20731	5.46312
C	0.34489	-0.30746	3.62502
C	2.65400	-4.40318	-0.33377
C	3.17961	-3.22861	2.43398
C	-3.29421	0.03587	0.57680
C	-2.82719	1.35922	0.34198
C	-3.21864	1.73548	-0.97721
C	-3.95191	0.65123	-1.54303
C	-3.99654	-0.39636	-0.58278
C	-3.08701	3.12333	-1.53259
C	-2.24649	2.29045	1.36442
C	-3.26430	-0.70301	1.88176
C	-4.87887	-1.60399	-0.66496
C	-4.77210	0.66596	-2.79979
H	-1.22470	-3.14241	-1.99502
H	-2.84110	-2.53834	-2.37600
H	-2.41733	-2.93774	-0.70937
H	-1.70823	1.75291	2.14884
H	-3.04309	2.86789	1.85362
H	-1.54696	3.00192	0.91899
H	-0.83385	3.30957	-3.44483
H	0.83601	3.14541	-3.98147
H	0.45245	3.08744	-2.25754
H	4.83317	1.60270	0.00219
H	5.18024	2.90091	1.15715
H	3.79240	3.03452	0.06288
H	-1.72983	-5.21444	2.95780
H	1.98581	-2.19084	-2.51690
H	1.82206	-2.35776	-4.26523
H	0.63078	-3.09678	-3.19489
H	2.58788	-0.03402	-1.64576
H	2.31807	1.66225	-2.02600
H	3.03041	0.59230	-3.23530
H	2.17269	-4.28402	-1.30776
H	2.13099	-5.18957	0.21350
H	3.68224	-4.74348	-0.50698
H	-4.56008	1.53844	-3.41961
H	-5.84185	0.69722	-2.55352
H	-4.61739	-0.22726	-3.41544
H	4.20882	-0.76118	0.16381
H	4.06023	-1.80201	-1.25747
H	5.10401	-2.29094	0.07862
H	4.10953	-3.80929	2.44581
H	2.38804	-3.84156	2.87392
H	3.31594	-2.35136	3.06919

H	-3.03186	0.59619	-5.03842
H	-1.75212	1.47931	-5.86274
H	-2.54166	2.19018	-4.46056
H	3.71983	-0.12070	3.83032
H	5.10110	0.92810	3.50702
H	4.78051	-0.44710	2.44610
H	-4.23949	-0.63551	2.38295
H	-2.52113	-0.29286	2.56790
H	-3.03717	-1.76647	1.76007
H	-0.96471	-1.76652	5.36365
H	-3.22006	3.14997	-2.61698
H	-2.11224	3.56529	-1.30631
H	-3.84813	3.78742	-1.10050
H	-4.97249	-1.98335	-1.68616
H	-5.89130	-1.35582	-0.31666
H	-4.51496	-2.42466	-0.04191
H	-1.37354	-2.83800	-4.37655
H	-1.19860	-1.86754	-5.84163
H	-2.61535	-1.66707	-4.81154
H	1.89410	3.53358	2.47413
H	3.27154	3.41719	3.58305
H	1.84543	2.39532	3.83070
H	0.01477	0.05992	4.60118
H	1.43835	-0.31911	3.62090
H	0.03998	0.41253	2.86276
H	0.06606	-5.35790	0.91479
H	-1.55802	-4.84995	0.46828
H	-0.16778	-3.92630	-0.10338
H	-1.57895	-4.15895	6.38417
H	-3.11801	-3.69343	5.66280
H	-2.39891	-5.25805	5.26461

105

From 2 - N-P coupling product

C	0.51184	-0.22020	-3.28472
C	0.89860	1.11316	-2.96520
C	-0.13073	1.99000	-3.40886
C	-1.15972	1.20092	-3.99579
C	-0.76100	-0.16857	-3.92033
C	2.22725	1.54212	-2.42045
U	-1.22590	0.52647	-1.30542
C	-2.02658	-1.75946	-1.31257
C	-0.03378	3.48752	-3.39079
C	-2.35350	1.69231	-4.76179
C	-1.48323	-1.30419	-4.58411
C	1.36370	-1.43924	-3.08309
N	0.48499	0.40110	0.11573
N	1.32668	1.02313	0.77228
N	2.23798	0.32273	1.54854
P	2.28494	-1.44321	1.24197
Si	4.09514	-2.44783	2.25403
C	5.71643	-1.78596	1.51335
Si	3.44888	1.57136	2.11860

C	4.67839	1.91822	0.72743
C	4.38571	1.04534	3.67103
C	2.51414	3.15116	2.55752
C	0.91244	-2.21782	2.22651
C	0.22692	-3.28437	1.59103
C	-0.80562	-3.94100	2.26689
C	-1.20107	-3.57833	3.55354
C	-0.53121	-2.51583	4.15887
C	0.51299	-1.83178	3.52825
C	0.56307	-3.74425	0.19561
C	-2.29346	-4.32376	4.27281
C	1.14805	-0.68994	4.27287
C	3.91478	-4.23345	1.62504
C	4.19614	-2.59697	4.14698
C	-2.48228	1.34662	1.00293
C	-2.20010	2.53472	0.27413
C	-3.03933	2.55630	-0.88073
C	-3.82579	1.36496	-0.86551
C	-3.49091	0.62583	0.30294
C	-3.27459	3.74640	-1.76523
C	-1.25134	3.61655	0.70253
C	-1.92432	0.98017	2.34640
C	-4.20972	-0.58797	0.81016
C	-4.93113	1.01515	-1.81995
H	-1.34255	-2.43322	-1.84526
H	-3.00613	-1.84701	-1.80836
H	-2.14264	-2.16066	-0.29676
H	-0.33163	3.20218	1.12706
H	-1.70285	4.26650	1.46392
H	-0.96507	4.26031	-0.13520
H	-0.97470	3.96258	-3.67624
H	0.73137	3.83425	-4.09784
H	0.25092	3.87838	-2.40675
H	5.21648	1.01655	0.42203
H	5.41694	2.66167	1.04777
H	4.15709	2.31318	-0.14903
H	-1.31916	-4.75778	1.76301
H	1.85735	-1.43575	-2.10555
H	2.14901	-1.49940	-3.84845
H	0.77606	-2.35854	-3.15239
H	2.70776	0.74046	-1.85420
H	2.13574	2.40452	-1.75246
H	2.90951	1.82938	-3.23259
H	3.92933	-4.28739	0.53286
H	2.99445	-4.70031	1.98727
H	4.75881	-4.82279	2.00255
H	-4.83934	1.55127	-2.76864
H	-5.91246	1.27449	-1.40043
H	-4.95662	-0.05670	-2.04467
H	5.99204	-0.78739	1.85918
H	5.64672	-1.76199	0.42110
H	6.53233	-2.46784	1.77930

H	4.98652	-3.31680	4.39154
H	3.25400	-2.99185	4.53938
H	4.42098	-1.66369	4.66633
H	-3.26381	1.13137	-4.52376
H	-2.19206	1.58485	-5.84279
H	-2.55667	2.74888	-4.57243
H	3.71901	0.91345	4.52714
H	5.07087	1.86703	3.91297
H	4.98942	0.14525	3.55387
H	-2.62869	1.24952	3.14505
H	-0.98768	1.50644	2.54470
H	-1.72331	-0.09132	2.43784
H	-0.82549	-2.20404	5.15959
H	-3.61445	3.46407	-2.76499
H	-2.38085	4.36491	-1.87976
H	-4.05316	4.39157	-1.33549
H	-4.56383	-1.22851	-0.00220
H	-5.08783	-0.29515	1.40202
H	-3.57369	-1.19930	1.45631
H	-1.20456	-2.27190	-4.16090
H	-1.25356	-1.33831	-5.65784
H	-2.57036	-1.21243	-4.49264
H	2.10017	3.62986	1.66832
H	3.19972	3.85358	3.04582
H	1.69257	2.94591	3.25024
H	0.84727	-0.69977	5.32453
H	2.23776	-0.73838	4.22894
H	0.85622	0.27334	3.84365
H	1.51247	-4.28797	0.15776
H	-0.21675	-4.40923	-0.18516
H	0.65394	-2.89305	-0.48343
H	-1.88825	-5.18157	4.82425
H	-2.80592	-3.68442	4.99778
H	-3.04040	-4.71193	3.57377

105

From 2 - N-N activation TS

C	-2.81673	3.31785	15.14310
C	-2.22285	2.50200	14.14315
C	-2.74242	1.19466	13.97882
C	-3.75740	0.71567	14.81286
C	-4.31678	1.49474	15.82017
C	-3.83750	2.79644	15.94682
P	-0.84696	3.10618	12.98156
Si	0.84407	1.48804	13.24430
C	0.86527	-0.01154	12.05313
C	-2.25332	0.24715	12.91677
C	-5.38609	0.95805	16.73296
C	-2.42564	4.74462	15.43107
N	-0.14460	4.44769	13.49644
Si	0.88115	5.52611	14.35073
C	1.63643	4.76182	15.92981
C	0.80939	0.87444	15.04086

C	2.48919	2.37469	12.93381
C	0.00233	7.12029	14.91669
C	2.31259	6.15454	13.26182
N	-1.19632	6.10476	11.89956
N	-1.68356	5.37681	11.19763
U	-2.40307	3.64034	9.65690
C	-2.48666	1.22996	9.47503
C	-1.05598	3.37624	7.31133
C	-0.06715	3.14663	8.31168
C	0.19298	4.38334	8.97088
C	-0.63491	5.37799	8.37599
C	-1.40258	4.76123	7.34650
C	0.71761	1.88425	8.51583
C	-1.51280	2.39797	6.26770
C	-2.12144	5.50310	6.25904
C	-0.54255	6.85741	8.61111
C	1.28173	4.62789	9.97721
C	-5.06775	3.12795	9.28058
C	-4.85453	4.38325	8.63455
C	-4.57331	5.35166	9.64090
C	-4.63146	4.70011	10.90962
C	-4.93492	3.32752	10.68320
C	-5.20571	4.65457	7.20109
C	-5.58380	1.89204	8.60530
C	-5.25389	2.32209	11.74761
C	-4.61737	5.37147	12.25116
C	-4.42266	6.83062	9.43412
H	-1.52120	0.74786	9.67935
H	-2.77094	0.94627	8.45052
H	-3.22444	0.77161	10.14473
H	-4.07004	6.31686	12.23560
H	-4.17254	4.73885	13.02347
H	-5.64344	5.59786	12.57015
H	-1.50953	7.35725	8.50096
H	0.13859	7.31680	7.88220
H	-0.15993	7.09360	9.60624
H	2.92084	5.34762	12.84423
H	2.97276	6.80613	13.84648
H	1.92173	6.74577	12.42689
H	-4.12477	-0.29731	14.65269
H	0.93832	1.69827	9.57037
H	1.68056	1.94550	7.99136
H	0.19331	1.00779	8.12816
H	2.10246	3.92043	9.82407
H	0.96024	4.54045	11.02173
H	1.69941	5.63187	9.85568
H	0.56130	-0.93216	12.55925
H	1.88945	-0.15479	11.69170
H	0.22039	0.11330	11.17984
H	-4.84544	5.62631	6.86062
H	-6.29736	4.65509	7.08109
H	-4.81634	3.89418	6.51650

H	2.56099	2.74076	11.90617
H	3.30317	1.65696	13.09235
H	2.63991	3.21825	13.60675
H	0.88532	1.71954	15.73043
H	1.65804	0.20499	15.22399
H	-0.11184	0.33387	15.27422
H	-2.84364	4.87697	5.73178
H	-1.39781	5.85623	5.51156
H	-2.64703	6.38724	6.62993
H	2.31742	3.93200	15.71729
H	0.85498	4.38709	16.59971
H	2.20434	5.52350	16.47735
H	-5.02145	1.30100	11.43359
H	-6.32617	2.34885	11.98352
H	-4.71420	2.51352	12.67733
H	-4.27330	3.44679	16.70388
H	-5.38165	7.34405	9.58357
H	-4.08644	7.07129	8.42182
H	-3.71011	7.27496	10.13535
H	-5.16998	1.76490	7.60006
H	-6.67591	1.94080	8.49874
H	-5.35226	0.98726	9.17163
H	-2.57618	2.50900	6.02897
H	-1.35381	1.36375	6.58183
H	-0.95992	2.54282	5.33022
H	0.75695	7.83037	15.27598
H	-0.70840	6.95744	15.73116
H	-0.53143	7.59333	14.08773
H	-3.22135	5.24203	15.99476
H	-1.51723	4.78399	16.03981
H	-2.21460	5.31634	14.52953
H	-1.85862	0.76213	12.04003
H	-1.46652	-0.41008	13.30039
H	-3.06702	-0.40177	12.57810
H	-5.96157	0.16110	16.25213
H	-4.94980	0.53788	17.64788
H	-6.08368	1.74375	17.03935

105

From 2 - N-N activation product

C	-5.18126	3.43183	10.27511
C	-5.30083	3.41518	8.85700
C	-4.95139	4.71016	8.36979
C	-4.60208	5.51995	9.48872
C	-4.75438	4.73166	10.66888
C	-5.92707	2.33045	8.03233
C	-5.24318	5.18227	6.97579
C	-4.31327	6.99252	9.46225
C	-4.71104	5.23272	12.08236
C	-5.61834	2.34368	11.20831
U	-2.60307	3.62124	9.33412
C	-2.93370	1.29353	8.77129
N	-1.77508	5.09606	11.10666

N	-1.22775	5.65683	11.90823
N	-0.34072	3.64063	13.20285
Si	0.77067	4.41528	14.25634
C	2.32938	5.01153	13.33560
P	-1.19204	2.48647	12.49180
Si	0.29447	0.65573	12.51109
C	2.03407	1.38748	12.34579
C	-2.64631	1.87635	13.55623
C	-3.17813	2.61925	14.64315
C	-4.25880	2.10695	15.37180
C	-4.85571	0.88172	15.08776
C	-4.35573	0.17910	13.99575
C	-3.28728	0.65518	13.23031
C	-2.65863	3.95571	15.10828
C	-2.87760	-0.19571	12.05883
C	-5.98553	0.34303	15.92312
C	0.17856	-0.65347	11.11469
C	0.15427	-0.19917	14.20106
C	1.33697	3.31513	15.71133
C	0.08468	6.01075	15.04484
C	-1.17785	3.50112	7.02160
C	-0.24693	3.12566	8.03235
C	0.05311	4.27885	8.81343
C	-0.69660	5.36906	8.28678
C	-1.45359	4.89401	7.17694
C	-1.62826	2.65637	5.86508
C	0.46009	1.80893	8.15793
C	1.11768	4.35982	9.87024
C	-0.54327	6.81303	8.66693
C	-2.07679	5.77359	6.13397
H	-2.01574	0.70203	8.89251
H	-3.23379	1.20057	7.71729
H	-3.71486	0.80368	9.36515
H	-4.09914	6.13201	12.18420
H	-4.32178	4.48078	12.77356
H	-5.72312	5.49041	12.42144
H	-1.48069	7.36886	8.57335
H	0.18641	7.30494	8.00990
H	-0.18941	6.93493	9.69287
H	2.85310	4.21223	12.80454
H	3.03287	5.45943	14.04750
H	2.06598	5.78320	12.60445
H	-4.81536	-0.76690	13.71217
H	0.59975	1.51324	9.20125
H	1.45910	1.86960	7.70563
H	-0.07522	1.00238	7.65176
H	1.98596	3.76086	9.57454
H	0.79941	4.01534	10.86143
H	1.46665	5.38894	9.98984
H	-0.16799	-1.61595	11.50280
H	1.17803	-0.80738	10.69378
H	-0.48600	-0.37018	10.29551

H	-4.77748	6.14349	6.75340
H	-6.32632	5.31250	6.84887
H	-4.92244	4.46854	6.21035
H	2.16406	1.91729	11.39790
H	2.75972	0.56567	12.37435
H	2.26583	2.07829	13.15579
H	0.29948	0.52841	15.00392
H	0.92584	-0.97236	14.29443
H	-0.82262	-0.66737	14.34814
H	-2.83486	5.25577	5.54327
H	-1.30405	6.11668	5.43226
H	-2.53452	6.67055	6.55954
H	1.92268	2.44938	15.38646
H	0.47830	2.94145	16.27955
H	1.96155	3.89705	16.39959
H	-5.48492	1.34870	10.77533
H	-6.68648	2.45135	11.44022
H	-5.07814	2.36612	12.15721
H	-4.64435	2.70213	16.19836
H	-5.22295	7.57079	9.67129
H	-3.94426	7.32008	8.48630
H	-3.57158	7.28218	10.21254
H	-5.49711	2.27071	7.02771
H	-7.00271	2.51636	7.91055
H	-5.81740	1.34725	8.49481
H	-2.66941	2.85114	5.58598
H	-1.54157	1.58935	6.08252
H	-1.01685	2.85996	4.97607
H	0.91006	6.54664	15.52902
H	-0.67867	5.82831	15.80568
H	-0.34217	6.67348	14.28681
H	-3.43712	4.48324	15.66882
H	-1.80458	3.82478	15.78011
H	-2.31223	4.58614	14.29245
H	-2.46941	0.39081	11.23480
H	-2.12386	-0.93624	12.34421
H	-3.73572	-0.75354	11.67147
H	-6.64396	-0.30612	15.33764
H	-5.60606	-0.25196	16.76336
H	-6.59186	1.14989	16.34614

103

From 2 - SiMe3 trans. adduct

C	0.04278	0.01910	-3.77253
C	1.28062	-0.10933	-3.08420
C	1.67426	1.18228	-2.63282
C	0.67520	2.11211	-3.04092
C	-0.32876	1.39079	-3.74633
C	2.14551	-1.33299	-3.03177
C	3.03337	1.49623	-2.08169
C	0.79488	3.61010	-3.04948
C	-1.35431	2.01868	-4.64296
C	-0.61572	-1.03153	-4.61671

U	-0.48150	0.43301	-0.99728
C	-0.87973	-1.88982	-1.46692
N	0.94854	0.21772	0.80058
Si	1.44349	1.83697	1.44953
C	3.30970	2.19243	1.52605
P	1.96920	-0.82321	1.81458
C	1.06292	-1.50954	3.29242
C	-0.12411	-2.27802	3.23578
C	-0.72670	-2.73441	4.40843
C	-0.18529	-2.47052	5.66745
C	1.00110	-1.74460	5.71719
C	1.63873	-1.26349	4.56652
C	-0.74837	-2.63485	1.92331
C	-0.86949	-2.94214	6.92228
C	2.95011	-0.54421	4.77387
Si	2.99577	-2.67411	0.92373
C	2.12291	-4.01090	-0.11176
C	3.66163	-3.53387	2.48280
C	4.50906	-2.02315	-0.02650
C	0.75668	2.15704	3.18876
C	0.77692	3.22573	0.30866
C	-3.16433	-0.18662	-0.29542
C	-2.68564	0.62248	0.76946
C	-2.49721	1.94250	0.26387
C	-2.86615	1.95048	-1.11150
C	-3.26238	0.62910	-1.46096
C	-3.08378	3.20216	-1.91134
C	-2.36406	3.17174	1.11627
C	-2.64216	0.25921	2.22715
C	-3.75682	-1.55885	-0.18002
C	-3.91486	0.17953	-2.73581
H	0.04511	-2.37177	-1.80880
H	-1.61002	-2.00757	-2.27892
H	-1.25704	-2.47283	-0.62220
H	-1.64407	3.05317	1.92830
H	-3.33266	3.41007	1.57847
H	-2.06520	4.04760	0.53468
H	-0.07984	4.12161	-2.63356
H	0.91261	3.97258	-4.07915
H	1.66890	3.95397	-2.49233
H	0.51637	4.07537	0.94940
H	-0.11623	3.03705	-0.29886
H	1.56847	3.55396	-0.36627
H	-1.63941	-3.32325	4.33317
H	2.42903	-1.60918	-2.01236
H	3.07707	-1.17416	-3.59088
H	1.64607	-2.19733	-3.47581
H	3.07841	2.48122	-1.61176
H	3.78337	1.49026	-2.88502
H	3.35401	0.76311	-1.33651
H	1.43791	-4.60137	0.50359
H	2.88729	-4.69290	-0.50396

H	1.56125	-3.61159	-0.95795
H	-3.92297	0.96756	-3.49137
H	-4.96085	-0.09912	-2.55295
H	-3.42881	-0.69880	-3.17557
H	4.25568	-1.46674	-0.93145
H	5.14532	-2.86563	-0.32149
H	5.09583	-1.36474	0.62103
H	4.29875	-2.86684	3.06952
H	4.26942	-4.39381	2.17760
H	2.85530	-3.89529	3.12630
H	-2.25203	1.40652	-4.75743
H	-0.93433	2.15027	-5.65057
H	-1.65925	3.00793	-4.29631
H	3.45783	3.27976	1.54685
H	3.84307	1.79673	0.65887
H	3.76823	1.76922	2.42188
H	-3.05415	-0.73731	2.40261
H	-3.24777	0.96396	2.81089
H	-1.63324	0.27058	2.64982
H	1.45699	-1.54229	6.68495
H	-3.38543	2.98243	-2.93630
H	-2.19733	3.84440	-1.96040
H	-3.88502	3.80392	-1.46248
H	-3.62312	-2.14322	-1.09392
H	-4.83732	-1.48718	0.00567
H	-3.32535	-2.13429	0.64194
H	-0.42051	-2.04215	-4.25056
H	-0.24776	-0.98014	-5.65052
H	-1.70161	-0.90388	-4.66001
H	-0.33327	2.09039	3.22901
H	1.04744	3.16358	3.51283
H	1.15150	1.44305	3.91460
H	3.79036	-1.24803	4.73424
H	3.13590	0.20490	4.00288
H	2.97623	-0.06687	5.75838
H	-1.59905	-3.30969	2.05562
H	-1.09773	-1.74221	1.40064
H	-0.03015	-3.11818	1.25686
H	-0.16832	-3.01641	7.75852
H	-1.66427	-2.24770	7.22190
H	-1.33484	-3.92271	6.78037

103

From 2 - SiMe3 trans. TS

C	0.42787	-0.45599	-3.37524
C	1.66193	-0.56795	-2.67740
C	2.05417	0.73513	-2.26026
C	1.04985	1.65304	-2.68208
C	0.05385	0.91565	-3.38335
C	2.53447	-1.78645	-2.62299
C	3.42243	1.08028	-1.74308
C	1.15401	3.15180	-2.66208
C	-0.99660	1.50675	-4.27673

C	-0.22709	-1.53447	-4.18675
U	-0.14545	0.03551	-0.61215
C	-0.69201	-2.28677	-1.04247
N	1.12265	-0.36418	1.10774
Si	2.26252	1.31165	2.23351
C	4.12686	1.72784	2.39403
P	2.16459	-0.99948	2.26928
C	1.41103	-1.81911	3.76519
C	0.27717	-2.66183	3.67250
C	-0.29593	-3.18065	4.83401
C	0.23076	-2.92613	6.10108
C	1.38121	-2.14555	6.17357
C	1.98485	-1.59281	5.03879
C	-0.29761	-3.05970	2.34647
C	-0.42787	-3.46910	7.34041
C	3.26652	-0.82844	5.26421
Si	3.70982	-2.59128	1.56592
C	2.85717	-3.92442	0.52558
C	4.49168	-3.38200	3.10093
C	5.08877	-1.73252	0.59132
C	1.36169	1.76269	3.84461
C	1.72993	2.57607	0.91962
C	-2.84895	-0.35207	0.12097
C	-2.26652	0.34865	1.20920
C	-1.96368	1.67047	0.76917
C	-2.37375	1.79076	-0.58990
C	-2.90800	0.53571	-0.99393
C	-2.47493	3.09690	-1.32214
C	-1.65441	2.82424	1.68067
C	-2.19208	-0.11380	2.63468
C	-3.54240	-1.67971	0.18482
C	-3.62336	0.21956	-2.27474
H	0.21768	-2.83298	-1.33334
H	-1.39404	-2.38453	-1.88252
H	-1.13705	-2.82907	-0.20232
H	-0.97311	2.55078	2.48966
H	-2.57792	3.19131	2.15158
H	-1.21523	3.67264	1.14742
H	0.17266	3.62767	-2.74051
H	1.74919	3.51382	-3.51223
H	1.62806	3.53396	-1.75384
H	1.92910	3.58263	1.31101
H	0.66493	2.52560	0.67819
H	2.31031	2.45402	0.00628
H	-1.17562	-3.81493	4.73917
H	3.06620	-1.87826	-1.67260
H	3.29724	-1.75598	-3.41435
H	1.96192	-2.70632	-2.76767
H	3.57279	2.16261	-1.69389
H	4.19395	0.68669	-2.41787
H	3.63150	0.67247	-0.74851
H	2.38402	-4.67068	1.17048

```
H       3.59410     -4.43918     -0.10105
H       2.09160     -3.49857     -0.12684
H      -3.55459      1.03874     -2.99296
H      -4.69086      0.04381     -2.08694
H      -3.23822     -0.68382     -2.76186
H       4.72676     -1.21471     -0.29925
H       5.81910     -2.48317      0.26744
H       5.61189     -1.00593      1.22028
H       5.10815     -2.66635      3.65153
H       5.14151     -4.20461      2.78011
H       3.73754     -3.78837      3.78029
H      -1.83995      0.82992     -4.43239
H      -0.57248      1.71671     -5.26904
H      -1.39051      2.45266     -3.89803
H       4.24946      2.81066      2.51965
H       4.67073      1.44644      1.48591
H       4.60671      1.23019      3.24175
H      -2.52504     -1.14877      2.73938
H      -2.84063      0.49965      3.27420
H      -1.17960     -0.05839      3.04444
H       1.83403     -1.95461      7.14536
H      -2.71660      2.95909     -2.37780
H      -1.55415      3.68677     -1.26686
H      -3.27325      3.71650     -0.89104
H      -3.47995     -2.22335     -0.76133
H      -4.60843     -1.54396      0.41438
H      -3.12490     -2.32873      0.95904
H      -0.01888     -2.53184     -3.79181
H       0.13267     -1.51160     -5.22469
H      -1.31540     -1.42173     -4.22470
H       0.38526      1.28014      3.92571
H       1.20671      2.84747      3.86561
H       1.94768      1.48578      4.72312
H       4.07692     -1.51642      5.53051
H       3.58480     -0.27036      4.38339
H       3.16220     -0.12354      6.09578
H      -1.28070     -3.52336      2.46447
H      -0.38603     -2.20495      1.67330
H       0.34658     -3.78802      1.84169
H       0.27631     -3.53245      8.17500
H      -1.25646     -2.82447      7.65873
H      -0.84324     -4.46704      7.16888
103
Complex 3 '
C      -0.09398     -0.32954     -3.97265
C       1.12422     -0.36386     -3.24112
C       1.42309      0.96280     -2.82231
C       0.38611      1.81785     -3.29025
C      -0.54686      1.01813     -4.01008
C       2.04795     -1.54074     -3.14611
C       2.71579      1.41426     -2.20770
C       0.41009      3.31812     -3.20426
```

C	-1.65202	1.50348	-4.90170
C	-0.66750	-1.45434	-4.78140
U	-0.74285	0.19155	-1.24571
C	-1.47011	-2.08751	-1.68640
N	0.54048	-0.26760	0.39460
Si	2.12881	1.65554	2.40309
C	3.94040	1.93311	2.92636
P	1.53978	-0.50959	1.64562
C	0.84481	-1.45861	3.12767
C	-0.27642	-2.31754	2.98573
C	-0.80696	-2.94843	4.11450
C	-0.26234	-2.80321	5.38903
C	0.89130	-2.03533	5.49795
C	1.45837	-1.37724	4.40094
C	-0.92120	-2.64043	1.66777
C	-0.88798	-3.46355	6.58702
C	2.75459	-0.65680	4.67930
Si	3.30924	-1.94698	1.06713
C	2.46151	-3.31634	0.07492
C	4.18180	-2.75526	2.54704
C	4.63988	-1.05336	0.05568
C	0.92956	2.16426	3.78392
C	1.85798	2.78832	0.91525
C	-3.39603	0.06988	-0.23019
C	-2.66359	0.85517	0.70050
C	-2.31661	2.08058	0.06011
C	-2.87443	2.07005	-1.25054
C	-3.51591	0.81590	-1.43856
C	-3.03025	3.29215	-2.10600
C	-1.74591	3.29275	0.73714
C	-2.46965	0.56614	2.16104
C	-4.14003	-1.20184	0.05135
C	-4.35805	0.39669	-2.60886
H	-0.59737	-2.69346	-1.97691
H	-2.18633	-2.15586	-2.51825
H	-1.93199	-2.58807	-0.82814
H	-1.12285	3.02610	1.59403
H	-2.54530	3.94846	1.11170
H	-1.13215	3.89986	0.06300
H	-0.48123	3.75878	-3.65546
H	1.27539	3.72893	-3.74122
H	0.47251	3.69096	-2.17444
H	1.62426	3.79819	1.27016
H	1.02936	2.43346	0.29909
H	2.75419	2.84234	0.29363
H	-1.67346	-3.59300	3.97989
H	2.71595	-1.46643	-2.28490
H	2.68222	-1.61587	-4.04100
H	1.50432	-2.48671	-3.06221
H	2.74285	2.50229	-2.10180
H	3.56903	1.13164	-2.83868
H	2.88439	0.98643	-1.21555

H	1.98055	-4.02882	0.75191
H	3.19906	-3.86298	-0.52290
H	1.70304	-2.90833	-0.59633
H	-4.29520	1.11294	-3.43149
H	-5.41669	0.32733	-2.32508
H	-4.07275	-0.58640	-3.00130
H	4.25404	-0.59856	-0.85754
H	5.40701	-1.78251	-0.23018
H	5.13110	-0.27381	0.64617
H	4.86021	-2.07403	3.06682
H	4.78117	-3.58683	2.15706
H	3.47119	-3.16493	3.26918
H	-2.55690	0.89359	-4.82281
H	-1.33742	1.46457	-5.95380
H	-1.93054	2.53842	-4.69264
H	4.13302	3.01020	2.85500
H	4.64087	1.43736	2.24734
H	4.16874	1.62107	3.94650
H	-2.88640	-0.40547	2.43727
H	-2.98153	1.31846	2.77563
H	-1.41641	0.55955	2.45683
H	1.38735	-1.95032	6.46372
H	-3.28328	3.04863	-3.13961
H	-2.13283	3.91674	-2.11965
H	-3.84518	3.92043	-1.71905
H	-4.17631	-1.85574	-0.82431
H	-5.17823	-0.98565	0.33974
H	-3.69285	-1.77621	0.86685
H	-0.45336	-2.43080	-4.33934
H	-0.24893	-1.45640	-5.79771
H	-1.75439	-1.37559	-4.88338
H	-0.07087	2.32391	3.37389
H	1.26735	3.11119	4.22072
H	0.84247	1.42627	4.58304
H	3.38259	-1.26712	5.33568
H	3.33584	-0.46106	3.77986
H	2.59425	0.29744	5.19162
H	-1.68079	-3.41572	1.80179
H	-1.38843	-1.76468	1.21881
H	-0.19985	-2.99834	0.93125
H	-0.16509	-3.59510	7.39733
H	-1.71433	-2.85834	6.97959
H	-1.29924	-4.44567	6.33407

References

[1] P. J. Fagan, J. M. Manriquez, E. A. Maatta, A. M. Seyam, T. J. Marks, *J. Am. Chem. Soc.* **1981**, 103, 6650-6667.

[2] P. Rungthanaphatsophon, P. Huang, J. R. Walensky, *Organometallics* **2018**, 37, 1884-1891.

[3] P. Rungthanaphatsophon, T. J. Duignan, A. J. Myers, S. P. Vilanova, C. L. Barnes, J. Autschbach, E. R. Batista, P. Yang, J. R. Walensky, *Inorg. Chem.* **2018**, 57, 7270-7278.

[4] J. F. Janik, E. N. Duesler, W. F. McNamara, M. Westerhausen, R. T. Paine, *Organometallics* **1989**, 8, 506-514.

[5] M. APEX2 Suite, WI, **2006**.

[6] G. Sheldrick, *Acta Crystallogr. C* **2015**, 71, 3-8.

[7] L. J. B. O. V. Dolomanov, R. J. Gildea, J. A. K. Howard, H. Puschmann, *J. Appl. Crystallogr.* **2009**, 42, 339-341.

[8] M. J. Frisch, G. W. Trucks, H. B. Schlegel, G. E. Scuseria, M. A. Robb, J. R. Cheeseman, G. Scalmani, V. Barone, G. A. Petersson, H. Nakatsuji, X. Li, M. Caricato, A. V. Marenich, J. Bloino, B. G. Janesko, R. Gomperts, B. Mennucci, H. P. Hratchian, J. V. Ortiz, A. F. Izmaylov, J. L. Sonnenberg, Williams, F. Ding, F. Lipparini, F. Egidi, J. Goings, B. Peng, A. Petrone, T. Henderson, D. Ranasinghe, V. G. Zakrzewski, J. Gao, N. Rega, G. Zheng, W. Liang, M. Hada, M. Ehara, K. Toyota, R. Fukuda, J. Hasegawa, M. Ishida, T. Nakajima, Y. Honda, O. Kitao, H. Nakai, T. Vreven, K. Throssell, J. A. Montgomery Jr., J. E. Peralta, F. Ogliaro, M. J. Bearpark, J. J. Heyd, E. N. Brothers, K. N. Kudin, V. N. Staroverov, T. A. Keith, R. Kobayashi, J. Normand, K. Raghavachari, A. P. Rendell, J. C. Burant, S. S. Iyengar, J. Tomasi, M. Cossi, J. M. Millam, M. Klene, C. Adamo, R. Cammi, J. W.

Ochterski, R. L. Martin, K. Morokuma, O. Farkas, J. B. Foresman, D. J. Fox, Wallingford, CT, **2016**.

[9] (a) J. P. Perdew, J. A. Chevary, S. H. Vosko, K. A. Jackson, M. R. Pederson, D. J. Singh, C. Fiolhais, *Phys. Rev. B* 1992, 46, 6671-6687; (b) A. D. Becke, *J. Chem. Phys.* **1993**, 98, 5648-5652.

[10] (a) W. Küchle, M. Dolg, H. Stoll, H. Preuss, *J. Chem. Phys.* **1994**, 100, 7535-7542; (b) X. Cao, M. Dolg, H. Stoll, *J. Chem. Phys.* **2003**, 118, 487-496; (c) X. Cao, M. Dolg, *J. Mol. Struct.* **2004**, 673, 203-209.

[11] W. Küchle, M. Dolg, H. Stoll, H. Preuss, *Mol. Phys.* **1991**, 74, 1245-1263.

[12] (a) R. Ditchfield, W. J. Hehre, J. A. Pople, *J. Chem. Phys.* **1971**, 54, 724-728; (b) W. J. Hehre, R. Ditchfield, J. A. Pople, *J. Chem. Phys.* **1972**, 56, 2257-2261; (c) P. C. Hariharan, J. A. Pople, *Theor. Chem. Acc.* **1973**, 28, 213-222; (d) M. M. Francl, W. J. Pietro, W. J. Hehre, J. S. Binkley, M. S. Gordon, D. J. DeFrees, J. A. Pople, *J. Chem. Phys.* **1982**, 77, 3654-3665.

[13] A. E. Reed, L. A. Curtiss, F. Weinhold, *Chem. Rev.* **1988**, 88, 899-926.
